# Acid-sensitive photoswitches: towards catalytic on-demand release of stored light energy†

**DOI:** 10.1039/d4sc04973j

**Published:** 2024-09-25

**Authors:** Léa Chocron, Nicolò Baggi, Enrique Ribeiro, Vincent Goetz, Pei Yu, Keitaro Nakatani, Rémi Métivier

**Affiliations:** a Université Paris-Saclay, ENS Paris-Saclay, CNRS, PPSM 91190 Gif-sur-Yvette France keitaro.nakatani@ens-paris-saclay.fr remi.metivier@ens-paris-saclay.fr; b Université Paris-Saclay, CNRS, ICMMO 91400 Orsay France; c CNRS, PROMES 66100 Perpignan France

## Abstract

Photochromic compounds are promising for a variety of applications, including molecular solar thermal (MOST) energy storage. The energy release step and cyclability are critical issues to be addressed for the development of this technology. We report herein the synthesis and characterization of two diarylethene molecules featuring one (1) or two (2) pyridine groups as protonatable moieties. Upon UV irradiation, both molecules undergo a cyclization reaction from the open form (OF) to the closed form (CF). Both CF are stable for a few days in acetonitrile, and the addition of acid leads to a 600 (1) or 1500-fold (2) acceleration of the ring-opening reaction, even in catalytic amounts. A kinetic model is proposed to simulate the reaction, elucidating the contribution of each step to the kinetics and evidencing the importance of the kinetic control over the protonation thermodynamic equilibrium. Data fitting leads to the rates of elementary steps and turnover numbers (TON). Following a complete reaction cycle, neutralization of the acid by an equivalent amount of base allowed further cycles. This study represents a significant advancement in the cyclability and the control of the on-demand triggering of the energy-releasing ring-opening reaction of diarylethenes for future MOST applications.

## Abstract 2

Molecular solar thermal energy storage route using teralylenes with pyridine protonatable moieties for on-demand energy release achieved upon protonation with catalytic amounts of acid.

## Graphical abstract

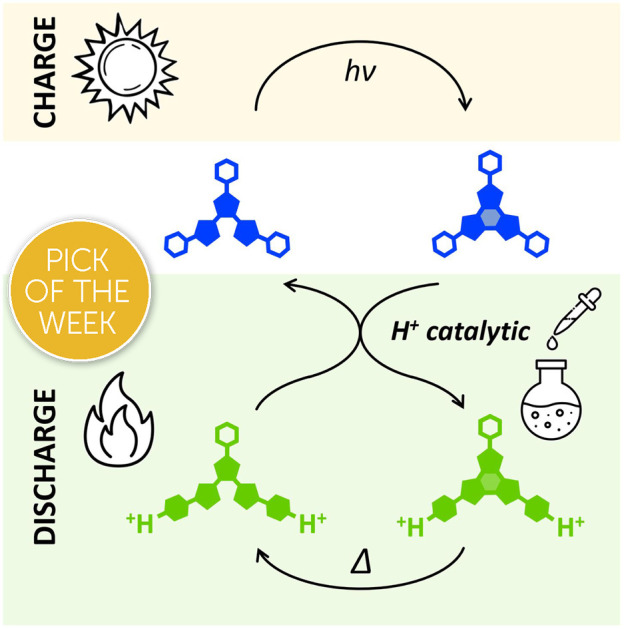

## Introduction

Photochromic molecules undergo reversible isomerization under light irradiation between a thermodynamically stable form and a metastable isomer, leading to a great variety of applications in chemistry, optics, mechanics or biological imaging.^1–5^ In particular, during the forward reaction, light energy can be chemically stored in the metastable isomer, and then released as heat during the back reaction.^6–8^ To ensure efficient storage, the design of MOlecular Solar Thermal (MOST) systems has to meet a certain number of criteria, as outlined by Moth-Poulsen and others,^6^ including a substantial energy difference between isomers of the photoswitchable molecule to maximize the stored solar energy combined with a long-term storage capacity, an efficient energy release and a good cyclability, facilitated by the use of an external triggering agent. The on-demand heat release is generally achieved by addition of a chemical catalyst, such as metal salts or metal complexes.^9–11^ In this work, we focus especially on an alternative method to release the stored energy while ensuring straightforward cyclability, with minimal amounts of protons as a simple triggering agent. In other words, we investigate the trigger of the back reaction of photochromic compounds using catalytic amounts of acid.

**Fig. 1:**
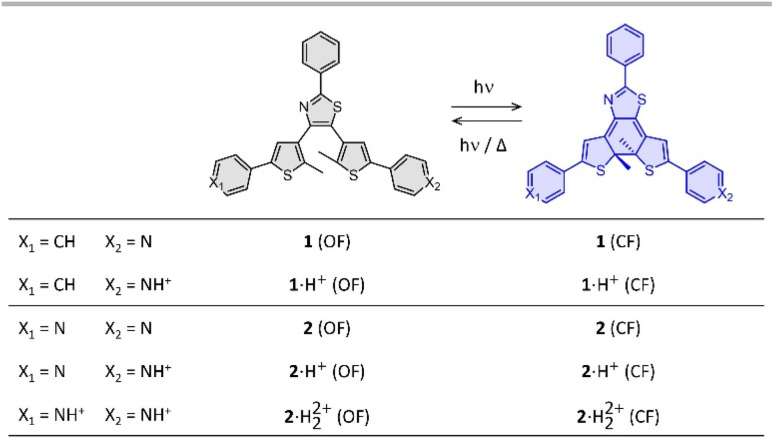
Photochromic reaction of the compounds studied between the open form (OF) and closed form (CF), in their neutral (1 and 2) and protonated (1·H^+^ and 2·H_2_^2+^) states.

In our study, we targeted terarylene-based derivatives, belonging to the diarylethene family of photochromic molecules,^1,3^ as they can be obtained through straightforward synthesis, without potentially harmful perfluorinated rings, often present in diarylethene structures. Moreover, they are widely recognized for their excellent fatigue resistance, high photoconversion^12^ and largely tunable thermal stability,^13–15^ which aligns with several criteria for the development of MOST systems.^16,17^ Indeed, the thermal stability of diarylethene is known to decrease upon incorporation of electron-withdrawing groups conjugated to the photochromic core.^17–20^ As part of our design strategy, we introduced either one or two pyridine moieties linked to the side-thiophenes and a simple phenyl substituent on the central thiazole ring, referred in Fig. 1 as compounds 1 and 2, respectively. In their neutral state, the pyridine moieties minimally impact the stability of the terarylenes. However, upon protonation, they turn into electron-withdrawing groups and consequently are expected to diminish the thermal stability of the closed form (CF).^21^ In this paper, we report on the impact of the acidity of the environment on the back reaction rates, to evaluate precisely the possibility to control the ring-opening reaction, especially under catalytic conditions, and we discuss the effect of the number of protonatable groups on the photoswitching properties.

## Results and discussion

Compounds 1 and 2 were conveniently prepared *via* palladium catalyzed cross-coupling reactions between suitable heterocyclic partners: Suzuki–Miyaura coupling followed by direct arylation for 1 and double Suzuki–Miyaura coupling for 2 (*cf.* Fig. S1 and detailed procedures in ESI†).^22–27^ The photochromic reaction of compounds 1 and 2 is depicted in Fig. 1, showing the conversion between the more stable open form (OF) and the metastable closed form (CF).

**Fig. 2:**
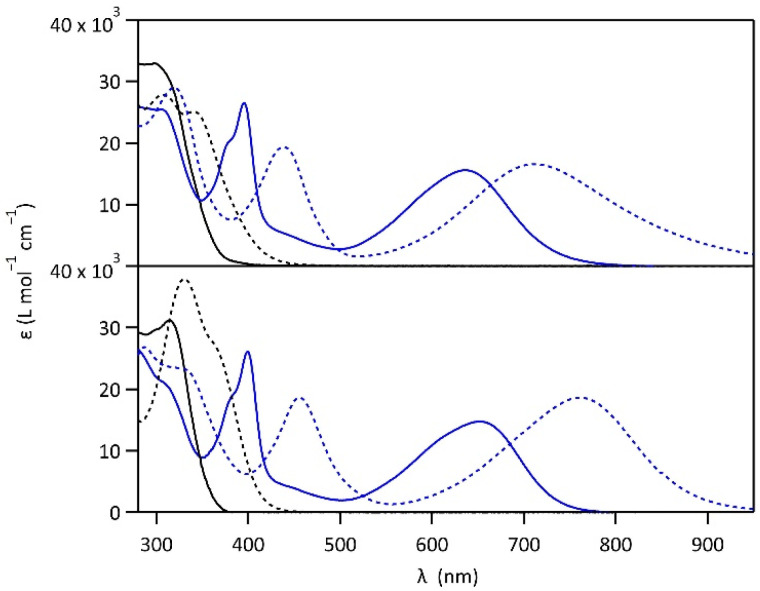
UV-vis spectra of 1 (top) and 2 (bottom) in the open forms (black curves) and closed forms (blue curves) in acetonitrile, in neutral (full lines) and acidic environment (in the presence of 10 eq. PTSA) (dotted lines).

The photochromic properties of both molecules were studied in acetonitrile at 20 °C under two conditions: pure acetonitrile (neutral environment) and in the presence of 10 equivalents of *para*-toluenesulfonic acid (PTSA) in acetonitrile (acidic environment). The UV-vis spectra of molecules 1 and 2 are shown in Fig. 2. The compounds in their OF absorb in the UV region with first band maxima at 300 nm (resp. 315 nm) for 1 (resp. 2) in neutral conditions, which are red-shifted to 307 nm and 340 nm (resp. 330 nm with a shoulder at 365 nm) for 1 (resp. 2) in acidic environment (Fig. 2). Upon irradiation with UV light (313 nm), colorless solutions in acetonitrile exhibited the growth of absorption bands in the visible region, associated with the corresponding CF, peaking at 635 nm (resp. 650 nm) for 1 (resp. 2) in neutral environment, and 710 nm (resp. 760 nm) for 1 (resp. 2) in acidic environment, corresponding to the S_0_ → S_1_ electronic transition. TD-DFT calculations, using the CAM-B3LYP functional and the 6-31G+(d,p) basis set, provided energy transitions well-consistent with the spectroscopy measurements (Fig. S3†).^28^

In neutral environment, 1 and 2 exhibit similar photo-isomerization properties, as listed in Table 1. High cyclization quantum yields (0.38 for 1 and 0.43 for 2, Fig. S2†) were measured under 313 nm light, leading to conversion yields close to unity at the photostationary state (PSS, with *α*_CF_ larger than 0.96 for both compounds). Much lower quantum yields were determined for the cycloreversion under irradiation at 640 nm (0.006 for both compounds), and close activation energy values for the thermal back reaction CF → OF were obtained (115 kJ mol^−1^ for 1 and 113 kJ mol^−1^ for 2, Fig. S5†), reflecting the good thermal stability of the closed isomer (*t*_1/2_ ∼ 25 days for 1 and 5 days for 2). Moreover, for both molecules, irradiation with white light (Xe-lamp), simulating the solar spectrum, leads to almost quantitative formation of the closed isomer (80% for 1 and 82% for 2, Fig. S10†) which is promising for solar energy storage applications. On the other side, when the pyridine moieties of 1 and 2 are protonated in acidic environment, the cyclization quantum yields at 313 nm are lowered (0.03 for 1·H^+^ and 0.37 for 2·H_2_^2+^, Fig. S2†). DFT calculations, providing HOMO and LUMO orbitals, give a possible explanation for the much lower photocyclization quantum yield of 1·H^+^ (0.03) compared to 1, 2 and 2·H_2_^2+^ (between 0.37 and 0.43). Indeed, the LUMO of 1·H^+^ shows a very limited bonding contribution on its reactive carbons, due to an electronic distribution localized exclusively on the protonated pyridine side of the molecule (Fig. S4†).

**Table 1:** Photochromic properties of compounds 1 and 2 in acetonitrile, in absence or presence of 10 eq. PTSA (neutral and acidic environments)

	*α*_CF_ a	*Φ*_OF→CF_ a	*Φ*_CF→OF_ b	*k*_CF→OF_ c [s^−1^]	*E*_a_ [kJ mol^−1^]/*A* [×10^12^ s^−1^]	Δ*H*_CF→OF_ [kJ mol^−1^]
1	0.96	0.38	0.006	4.5 × 10^−7^	115/85	84
1·H^+^	0.79^d^	0.03	n.d.^e^	2.2 × 10^−4^	90/1.8	86
2	0.97	0.43	0.006	2.2 × 10^−6^	113/120	113
2·H_2_^2+^	0.88^d^	0.37	n.d.^e^	2.8 × 10^−3^	93/80	118

a Irradiation at 313 nm.

b Irradiation at 640 nm.

c Temperature 20 °C.

d Irradiation power = 2 mW.

e Not determined.

**Fig. 3:**
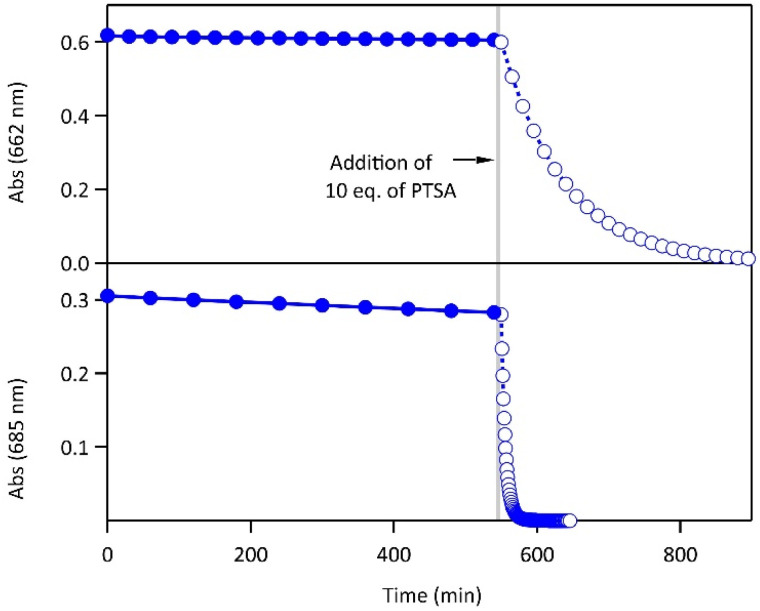
Evolution of the absorbance at 662 nm (resp. 685 nm) corresponding to the decay of the CF of 1 (top) and 2 (bottom) at 20 °C in acetonitrile (full circles, neutral environment) and upon addition of 10 eq. of PTSA (open circles, acidic environment), CF was produced beforehand by irradiating the OF at 313 nm. After reaching PSS, irradiation was stopped at time zero.

Most importantly, the activation energies of the thermal ring-opening are much lower in acidic environment (90 kJ mol^−1^ for 1·H^+^ and 93 kJ mol^−1^ for 2·H_2_^2+^, Fig. S5†). These results reveal a clear difference in photochromic behavior of both compounds 1 and 2 between the neutral and acidic environments, especially concerning the thermal stability of the closed isomer. While the CFs of both molecules display relatively high thermal stability in their neutral forms at 20 °C (*t*_1/2_ in the range of several days, *cf.*Table 1), the thermal stability decreased upon protonation, resulting in *t*_1/2_ of 1 h for the monoprotonated derivative 1·H^+^(CF) and 5 min for 2·H_2_^2+^(CF) (Fig. 3). Thus, the protonation enhances the thermal back reaction rate by a factor 600 and 1500, respectively, for 1 and 2 compared with a neutral medium. Such an acceleration of the spontaneous cycloreversion reaction at room temperature has been reported only a few times in literature.^29,30^ The addition of Et_3_N to the acidic solution enabled the recovery of the properties observed in neutral acetonitrile, demonstrating the chemical reversibility and the good cyclability of the system (Fig. S8†). Moreover, differential scanning micro-calorimetry experiments on the CF of 1 and 2 allowed the determination of the energy released during the thermal back reaction at 65 °C in neutral acetonitrile and at 25 °C in acidic acetonitrile.^31^ The Δ*H*_CF→OF_ values range between 84 and 120 kJ mol^−1^ (Table 1 and Fig. S9,† consistent with the DFT calculations listed in Table S1†), in line with the requirements established for potential MOST applications.^6^

Quantitative studies of the effect of the protonation properties in acetonitrile of the OF and CF of both molecules were determined by progressive addition of a 1.2 × 10^−2^ M solution of PTSA in acetonitrile to a solution of 1 or 2. Their absorption spectra with increasing amounts of acid are displayed in Fig. S6,† showing bathochromic shifts and clear isosbestic points, with no spectral changes above one (resp. two) equivalents of protons for 1 (resp. 2). This indicates that 1 and 2 behave, in this concentration range, as strong bases. Therefore, the protonation of the pyridine moieties with PTSA appears to be quasi quantitative. Moreover, no protonation on the nitrogen of the thiazole bridge occurs in these concentration ranges.

**Fig. 4:**
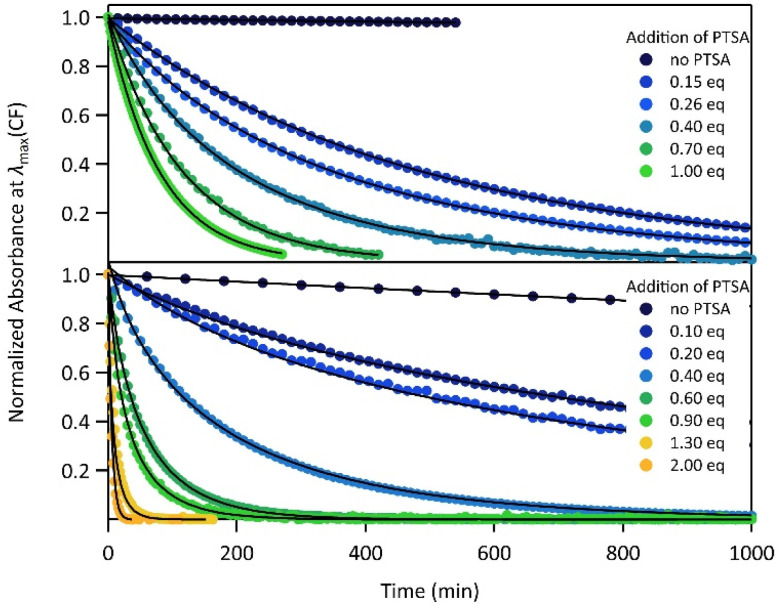
Kinetics of the thermal back reaction at 20 °C for 1 (top) and 2 (bottom) in the presence of different PTSA equivalents (full circles), and corresponding curve fits obtained from the kinetic analyses, based on the mechanism shown in Scheme 1 (black lines).

The proton-dependent thermal back reaction at 20 °C of both compounds was then investigated. Starting from the PSS, the absorption spectra were recorded right after addition of given amounts of PTSA (Fig. S7†). The time evolution of the normalized absorbance value (within the absorption band in the visible of CF) with different amounts of PTSA, is displayed in Fig. 4.

A clear acceleration of the ring-opening reaction was observed upon increasing amounts of acid. Remarkably, sub-stoichiometric amounts of acid are enough to induce a strong acceleration of the thermal CF → OF reaction, whereas above one equivalent of protons per pyridine unit (1 eq. for 1 and 2 eq. for 2), only poor influence of the concentration of acid on the thermal back reaction was observed. The times at which 10% of the OF is recovered, noted *t*_10%_, are listed in Table 2 for both molecules in the presence of different amounts of PTSA. These values reveal a real effect of catalytic quantities of protons on ring-opening kinetics, with, for example, an acceleration by a factor of ∼50 (resp. ∼10) for 1 (resp. 2), in the presence of only 0.1 equivalents of acid, compared to the neutral solution.

**Table 2:** Proton-catalyzed thermal back reaction parameters of 1 and 2 in acetonitrile

	Eq. H^+^ (PTSA)	*t*_10%_ [s]^a^	TON_max_^b^	TON_max·th_^c^
1	0	2.4 × 10^5^	—	—
	0.1	4.6 × 10^3^	8.8	10
	0.3	2.7 × 10^3^	3.3	3.3
	0.4	1.2 × 10^3^	2.5	2.5
	0.7	6.3 × 10^2^	1.4	1.4
2	0	4.8 × 10^4^	—	—
	0.1	4.5 × 10^3^	11.6	20
	0.2	2.7 × 10^3^	6.6	10
	0.4	6.5 × 10^2^	4.4	5
	0.6	3.5 × 10^2^	3.0	3.3
	0.9	50	2.1	2.2
	1.5	<30	1.3	1.3

a *t*_10%_: time at which 10% of the OF is recovered.

b TON_max_: maximum value of the experimental turnover number.

c TON_th_: theoretical maximum turnover number.

**Scheme 1:**
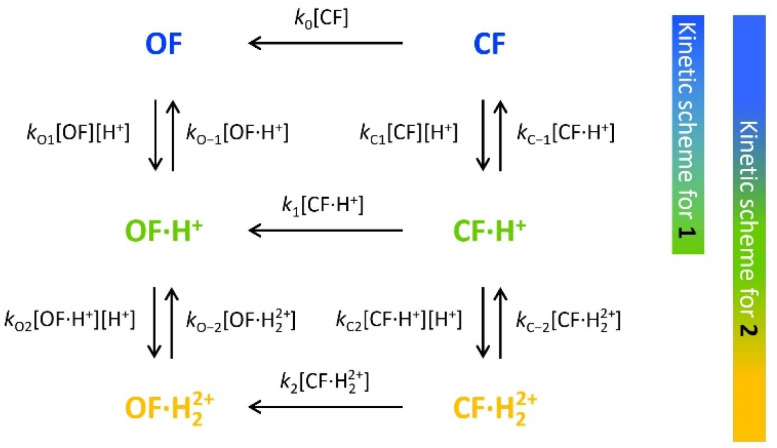
Elementary reactions governing the proton-catalyzed thermal back reaction mechanism of 1 and 2.

To characterize and model the catalytic effect of the protons in the presence of different acid equivalents, the absorbance curves recorded at different acid equivalents (Fig. 4) were fitted with kinetic equations corresponding to the elementary reactions presented on Scheme 1. Each absorption time-profile was fitted individually and the full list of kinetic rate constants of proton exchanges were appropriately determined. The average values of the rate constants, associated with their corresponding standard deviations, are displayed in Table 3. Moreover, in the case of compound 2, *k*_1_, the kinetic rate constant of the thermal back reaction of compound 2·H^+^(CF), which cannot be isolated, also appears as a fitting parameter and was found to be (2.0 ± 0.1) × 10^−4^ s^−1^. Additionally, we considered that the protonation rate constants of both pyridine units of 2(OF) were equivalent (*k*_O1_ = *k*_O2_ and *k*_O−1_ = *k*_O−2_). Even though the data are fitted independently, the low standard deviations indicate a good correlation of the kinetic model and the experiments ensure the reliability of the determined parameters.

**Table 3:** Kinetic (*k*_i_) and equilibrium (*K*_i_) constants determined by fitting the experimental back reaction kinetics at various PTSA equivalents in acetonitrile solution

	*k*_O1_ (=*k*_O2_) [×10^6^ s^−1^mol^−1^ L]	*k*_O−1_ (=*k*_O−2_) [×10^−1^ s^−1^]	*k*_C1_ [×10^6^ s^−1^mol^−1^ L]	*k*_C−1_ [×10^−1^ s^−1^]	*k*_C2_ [×10^6^ s^−1^mol^−1^ L]	*k*_C−2_ [×10^−1^ s^−1^]	*K*_O1_ (=*K*_O2_) [×10^7^]	*K*_C1_ [×10^7^]	*K*_C2_ [×10^7^]
1	1.7 ± 0.3	1.2 ± 0.2	1.8 ± 0.2	2.2 ± 0.2	—	—	1.5 ± 0.4	0.8 ± 0.1	—
2	1.8 ± 0.2	1.1 ± 0.1	2.0 ± 0.1	2.6 ± 0.4	1.9 ± 0.1	5.9 ± 0.1	1.7 ± 0.2	0.8 ± 0.1	0.35 ± 0.07

This modelling can be used to plot the concentration of each species as a function of time (Fig. S7†) and to deduce an important parameter of the proton's catalytic efficiency, its turnover number (TON), defined at every instant as below:
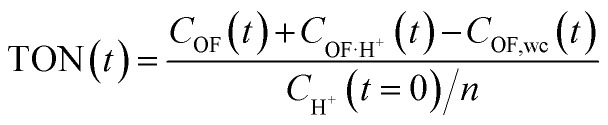
where *C*_OF_(*t*) and *C*_OF·H^+^_(*t*) are respectively the concentrations of OF and OF·H^+^ at a given time *t*, *C*_H^+^_(*t* = 0), the initial concentration of acid, *n* the number of pyridine units per molecule and *C*_OF wc_(*t*) the concentration of OF without catalysis calculated as *C*_tot_(1 − *e*^−*k*_0_*t*^). The TON curves as a function of time are displayed in Fig. S7.† All curves have a similar shape and reach a maximum value corresponding to the maximum efficiency of the proton, which then decreases due to noticeable effect of the spontaneous back reaction in the absence of a catalyst at long timescales.

These values of maximum TON, noted TON_max_ for simplicity, for each experiment are reported in Table 2, and compared with the theoretical maximum TON, noted TON_max·th_ when the spontaneous CF → OF reaction is negligible (*k*_0_ = 0). Thus, in most cases, the catalytic efficiency of the proton is very close to its theoretical maximum value, which testifies to the performance of the catalytic ring-opening compared to spontaneous opening, especially for compound 1, which features a slower spontaneous back reaction.

Concerning the global set fitting of parameters, several deductions can be drawn. Firstly, the 1·H^+^ compound serves as an effective model for the study of 2·H^+^. Indeed, both compounds demonstrate almost identical thermal stability in the CF: *k*_1_ of compound 1 was measured at 2.2 × 10^−4^ s^−1^ and *k*_1_ of compound 2 was fitted at 2.0 × 10^−4^ s^−1^. Additionally, analogous proton exchange rate constants *k*_O1_, *k*_O−1_, *k*_C1_ and *k*_C−1_ were obtained for both 1 and 2 (*cf.*Table 3). However, at equal proton equivalents below 1 equivalent, the back reaction of 2 is faster than 1 due to the impact of the double pyridine protonation that further accelerates the reaction. For example, at 0.4 acid equivalents, *t*_10%_ is measured at 1200 s for 1 and 650 s for 2. Secondly, the proton exchange rate constants of both molecules indicate fast direct protonation reactions (*k*_O1_, *k*_O2_, *k*_C1_ and *k*_C2_, determined as ∼2 × 10^6^ s^−1^ mol^−1^ L) and much lower deprotonation rate constants (*k*_O−1_, *k*_O−2_, *k*_C−1_ and *k*_C−2_, in the range of 0.1–0.6 s^−1^). Alongside the fit, the concentrations of every species can be calculated and it was found that less than 1% of H^+^ species remains in solution (Fig. S7†). Therefore, such values are well-compatible with the strong base behaviour previously identified for both OF and CF of compounds 1 and 2.

**Fig. 5:**
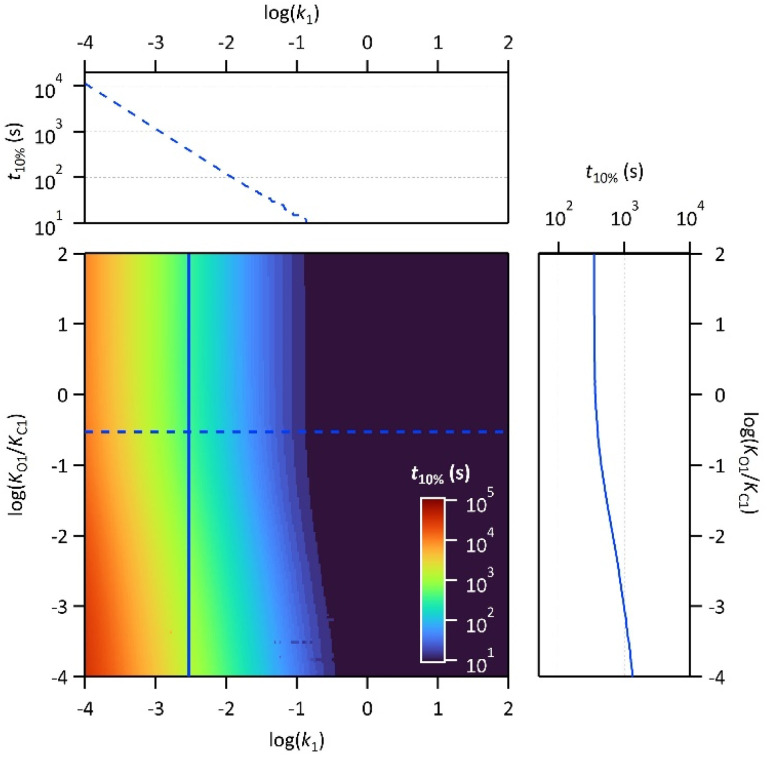
2D plot of the calculated parameter *t*_10%_ (time required to recover 10% of the OF) as a function of the thermal back reaction kinetic constant *k*_1_ and the ratio of the protonation equilibrium constants *K*_O1_/*K*_C1_. Horizontal and vertical cross-sections are plotted in blue: horizontal cross-section = dotted line for *K*_O1_/*K*_C1_ = 0.3; vertical cross-section = full line for *k*_1_ = 3 × 10^−3^ s^−1^.

Finally, the thermodynamic protonation constants of the OF, OF·H^+^, CF and CF·H^+^, namely *K*_O1_, *K*_O2_, *K*_C1_ and *K*_C2_, can be estimated from the ratio between the protonation and deprotonation kinetic rate constants. For both compounds, the same tendency is observed: the protonation of the OF is up to 5 times more favourable thermodynamically than the protonation of the CF (Table 3). Thus, over the course of the reaction, a competition takes place between the protonation of the OF that has been formed and the CF that has not yet disappeared. This phenomenon is observed experimentally with the hypsochromic shift in the visible absorption band of the CF while diminishing, reflecting the progressive deprotonation of the CF in favour of the OF (Fig. S7†). All these observations indicate that the driving force behind the catalytic effect lies in the rapid kinetics associated with the opening of the CF in its protonated form. Thus, even if the thermodynamics tends to disfavour the catalytic effect by preferential formation of OF·H^+^ compared to CF·H^+^, the kinetic effect seems to predominate in these experiments. Based on these results, numerical simulations were carried out to estimate the relative thermodynamic and kinetic effects in such a system driven by proton-catalyzed CF → OF reactions. To do so, the concentrations of the various species in the kinetic model shown in Scheme 1 were calculated, considering only the first protonation steps, in the presence of a fixed catalytic amount of acid, by varying the *K*_O1_/*K*_C1_ and *k*_1_ parameters. The parameter *t*_10%_ (time at which 10% of the OF is recovered) was chosen as an indicator of the catalyzed back reaction rate. The 2D plot of *t*_10%_ as a function of *K*_O1_/*K*_C1_ and *k*_1_ is shown in Fig. 5.

**Scheme 2:**
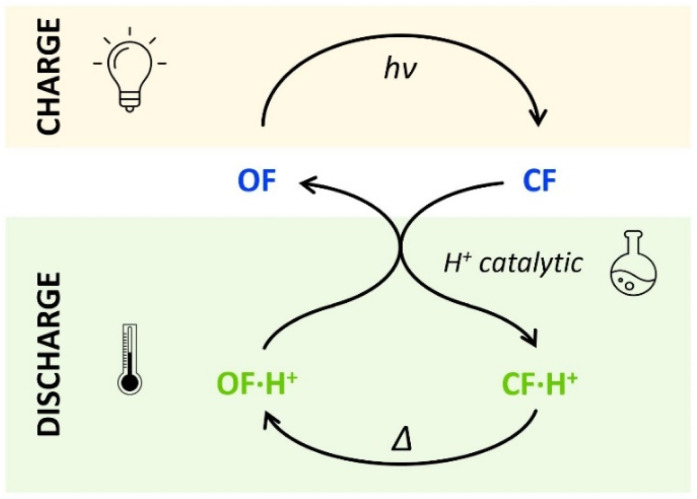
Charge (absorbing light energy) and discharge (releasing heat energy) principles, through acid-catalyzed triggered back reaction, demonstrated for compounds 1 and 2.

The horizontal and vertical cross-sections of the 2D plot of *t*_10%_, shown in Fig. 5 provide a better view of the kinetic and thermodynamic effects, respectively. For example, when *k*_1_ ranges between 10^−4^ and 10^2^ s^−1^, for *K*_O1_/*K*_C1_ fixed at 0.3 (dotted curve), the value of *t*_10%_ extends from 1.1 × 10^4^ s to 5 s (more than three orders of magnitude). In contrast, when *k*_1_ is fixed at 3 × 10^−3^ s^−1^ and *K*_O1_/*K*_C1_ spans between 10^−4^ and 10^2^, the value of *t*_10%_ varies only from 1.3 × 10^3^ s to 340 s (less than one order of magnitude). These numerical results agree with the experimental ones, demonstrating that the catalytic effect of protons is much more impacted by the kinetics than the thermodynamics of the system. In other words, when the CF·H^+^ → OF·H^+^ kinetic rate constant is fast enough, the proton-triggered back-reaction can proceed with high efficiency at catalytic amounts of protons, even if the thermodynamics is unfavourable. Scheme 2 summarizes this new concept of acid-sensitive MOST system, enabling to load energy under solar illumination, then to release the stored energy on-demand, with catalytic amounts of protons.

## Conclusion

In conclusion, we have studied two photochromic compounds belonging to the diarylethene family, terarylenes 1 and 2, possessing one and two protonatable pyridine groups, respectively. Both exhibit excellent photochromic properties in acetonitrile, with a very slow thermal recovery after irradiation (several days) making them essentially bistable in the neutral form. Interestingly, this thermal back reaction from the CF to the OF is significantly accelerated in the presence of acid, since the *t*_1/2_ at 20 °C is reduced to 1 h for 1 and even 5 min for 2, due to a substantial reduction of the activation energy of the CF → OF reaction. Even more noteworthy is the observation that a sub-stoichiometric amount of acid is sufficient to accelerate this thermal back reaction: indeed, the addition of 0.1 eq. of protons reduces the CF → OF time by more than one order of magnitude. We have developed a complete kinetic model involving all the elementary processes of protonation, deprotonation and ring opening of each species (neutral, mono and doubly protonated), which proves to perfectly reproduce the experimental observations. In the acetonitrile medium considered, protonation is extremely rapid and efficient, leading to a sufficient concentration of protonated CF·H^+^, which undergo a CF·H^+^ → OF·H^+^ reaction much faster than in the neutral state, giving rise to the overall effect of ring-opening acceleration process, despite the low proton concentration. This demonstrates the kinetic control of the catalytic triggering of the back reaction, even under unfavourable thermodynamic conditions. Given that terarylene systems 1 and 2 are also highly photoresistant, with excellent reversibility, cyclability, and satisfactory thermal energy release during the CF → OF reaction (in the range of 84–120 kJ mol^−1^), they represent a particularly original and promising family of photoswitches for MOST applications allowing the on-demand delivery of thermal energy by catalytic processes from solar energy.

## Data availability

The data supporting this article have been included as part of the ESI.†

## Author contributions

The authors confirm contribution to the paper as follows (CRediT): conceptualization: L. C., P. Y., K. N., R. M.; formal analysis: L. C.; funding acquisition: R. M.; investigation: L. C., N. B., E. R., P. Y.; methodology: L. C., V. G., P. Y., K. N., R. M.; project administration: K. N., R. M; resources: V. G., P. Y., K. N., R. M.; software: L. C.; supervision: K. N., R. M.; visualization: L. C., R. M.; writing – original draft: L. C.; writing – review & editing: L. C., N. B., E. R., V. G., P. Y., K. N., R. M. All authors reviewed the results and approved the final version of the manuscript.

## Conflicts of interest

There are no conflicts to declare.

## Supplementary Material

SC-OLF-D4SC04973J-s001
