# Supplementary material for: Acid-sensitive photoswitches: towards catalytic on-demand release of stored light energy
Source: Chem Sci. 2024 Sep 25;15(39):16034–9. doi: 10.1039/d4sc04973j (PMC11423652; doi:10.1039/d4sc04973j)
Supplement: SC-015-D4SC04973J-s001 [file SC-015-D4SC04973J-s001.pdf]

Supplementary Information (SI) for Chemical Science.
This journal is © The Royal Society of Chemistry 2024

Supporting Information

Acid-Sensitive Photoswitches: Towards Catalytic On-Demand Release of Stored Light Energy

Léa Chocron,[a] Nicolò Baggi,[b,†] Enrique Ribeiro,[c] Vincent Goetz,[c] Pei Yu,[b] Keitaro Nakatani*,[a] and Rémi Métivier*,[a]

a L. Chocron, Pr. K. Nakatani and Dr. R. Métivier
Université Paris-Saclay, ENS Paris-Saclay, CNRS, PPSM, 91190 Gif-sur-Yvette, France.
E-mail: keitaro.nakatani@ens-paris-saclay.fr; remi.metivier@ens-paris-saclay.fr

b Dr. N. Baggi and Dr. Pei Yu
Université Paris-Saclay, CNRS, ICMMO, 91400 Orsay, France.

c Dr. E. Ribeiro and Dr. V. Goetz
CNRS, PROMES, 66100 Perpignan, France.

† Present address: Dpt Chemical Engineering, Universitat Politècnica de Catalunya, EEBE, 08019 Barcelona, Spain.

* Corresponding authors

Table of contents

1. **Materials and synthetic protocols** 2
2. **Spectroscopy and photokinetic experiments** 9
3. **DFT calculations** 9
4. **Activation energy determination** 13
5. **Acidic titrations** 14
6. **Catalyzed back reaction** 15
7. **Cyclability experiments** 21
8. **Determination of reaction enthalpies** 22
9. **White light irradiations** 24

1. Materials and synthetic protocols

1.1 Materials for synthesis and synthetic routes for compounds **1** and **2**

Air-sensitive syntheses were performed under argon using standard Schlenk techniques. Chemicals and solvents were used as received unless otherwise stated.

Terarylenes **1** and **2** are prepared according to the synthetic routes shown in Figure S1. The starting compounds 2-methyl-3-bromo-5-phenylthiophene **7** [1], 2-methyl-3-bromo-5-(4-pyridyl)thiophene **8**[2], and 2-Ph-4,5-dibromo-thiazole **6** [3] were synthesized as described in the literature.

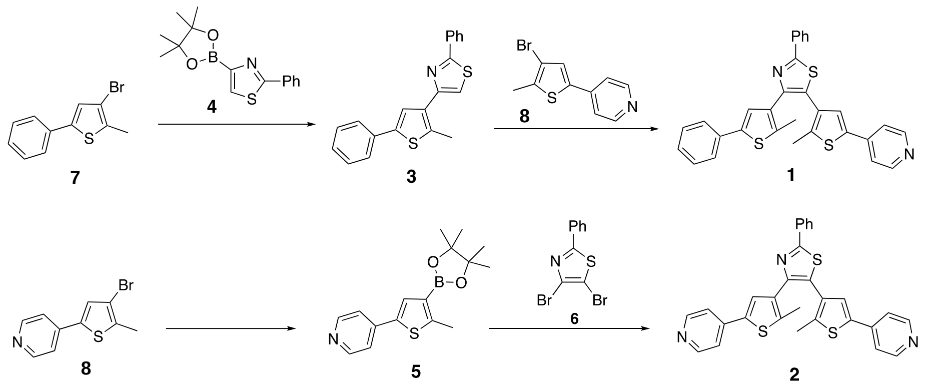

Figure S1: Syntheses of terarylenes **1** and **2**.

1.2 Synthetic protocols for **1**

(a) 2-phenyl-4-(4,4,5,5-tetramethyl-1,3,2-dioxaborolan-2-yl)thiazole (**4**)[4]

4 was prepared in a different way as reported4a via a standard Li/Br exchange followed by reaction with 2-isopropoxy-4,4,5,5-tetramethyl-1,3,2-dioxaborolane in diethyl ether as follows.

To a solution of 2-phenyl-4-bromo-thiazole[5] (480 mg, 2 mmol) in dry diethyl ether (c.a. 25 mL) at - 78°C was added dropwise *n*-BuLi (1 mL, 2.5 M in hexane). The resulting solution was kept at that temperature for c.a. 20 min. before neat 2-isopropoxy-4,4,5,5-tetramethyl-1,3,2-dioxaborolane (0.5 mL, 2.5 mmol) was added quickly. The solution was allowed to slowly warm back to RT overnight before aqueous NH_4Cl solution (20 mL, 1 M) was added. The aqueous phase was extracted twice with diethyl ether (20 mL each). The combined organic phase was washed with brine (20 mL), dried over Na_2SO_4 and filtered. After removal of solvents the residue slowly solidified on standing in air to give a weakly colored low-melting solid (540 mg, 94 % yield), which was used without further purification in the next step.
^{1}H-NMR (300 MHz, $CDCl_3$): δ (ppm) 8.05-8.02 (m, 2H), 7.97 (s, 1H), 7.44-7.39 (m, 3H), 1.39 (s, 12H).

^{1}H-NMR of **4**

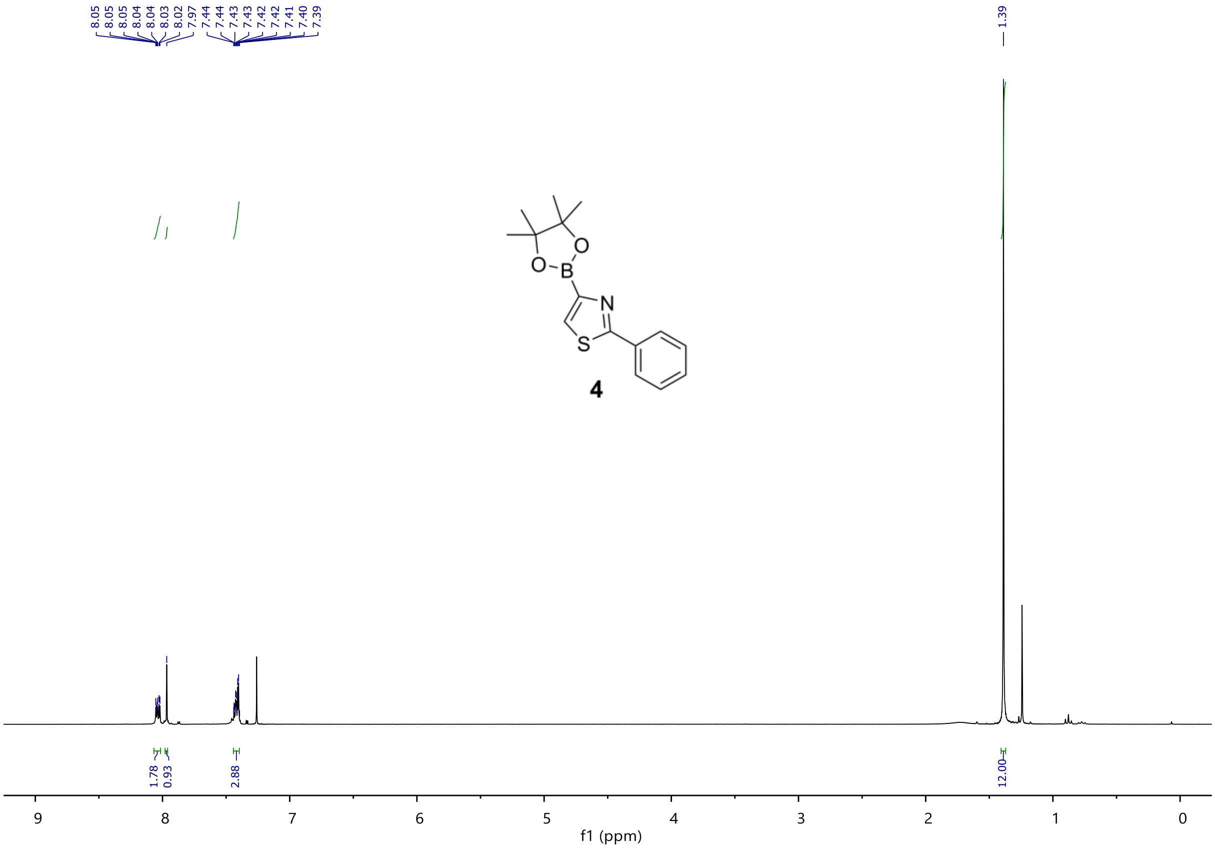

(b) Compound **3**

2-methyl-3-bromo-5-phenylthiophene **7** (253 mg, 1 mmol), compound **4** (301 mg, 1.05 mmol), CsF (427 mg, 2.8 mmol) and $Pd(PPh_3)_4$ (24 mg, 0.02 mmol) were partially solubilized in anhydrous dioxane (c.a. 15 mL) and the mixture was then refluxed overnight. At RT, water (c.a. 20 mL) and dichloromethane (c.a. 20 mL) were introduced. The organic phase was separated and washed with brine (c.a. 20 mL), dried over Na_2SO_4 and filtered. After removal of solvents the residue was purified by silica gel column chromatography (25 to 30% CH_2Cl_2/PE) to give **3** as a white crystalline solid (277 mg, 83 % yield). ^{1}H-NMR (300 MHz, $CDCl_3$): δ (ppm) 8.06-8.03 (m, 2H), 7.64-7.61 (m, 3H), 7.47-7.36 (m, 5H), 7.28 (m, 2H), 2.77 (s, 3H).HRMS (ESI): calcd for $C_{20}H_{15}NS_2.H^+$: 334.0719 $[M+H]^+$, found: 334.0709.

^{1}H-NMR of **3**

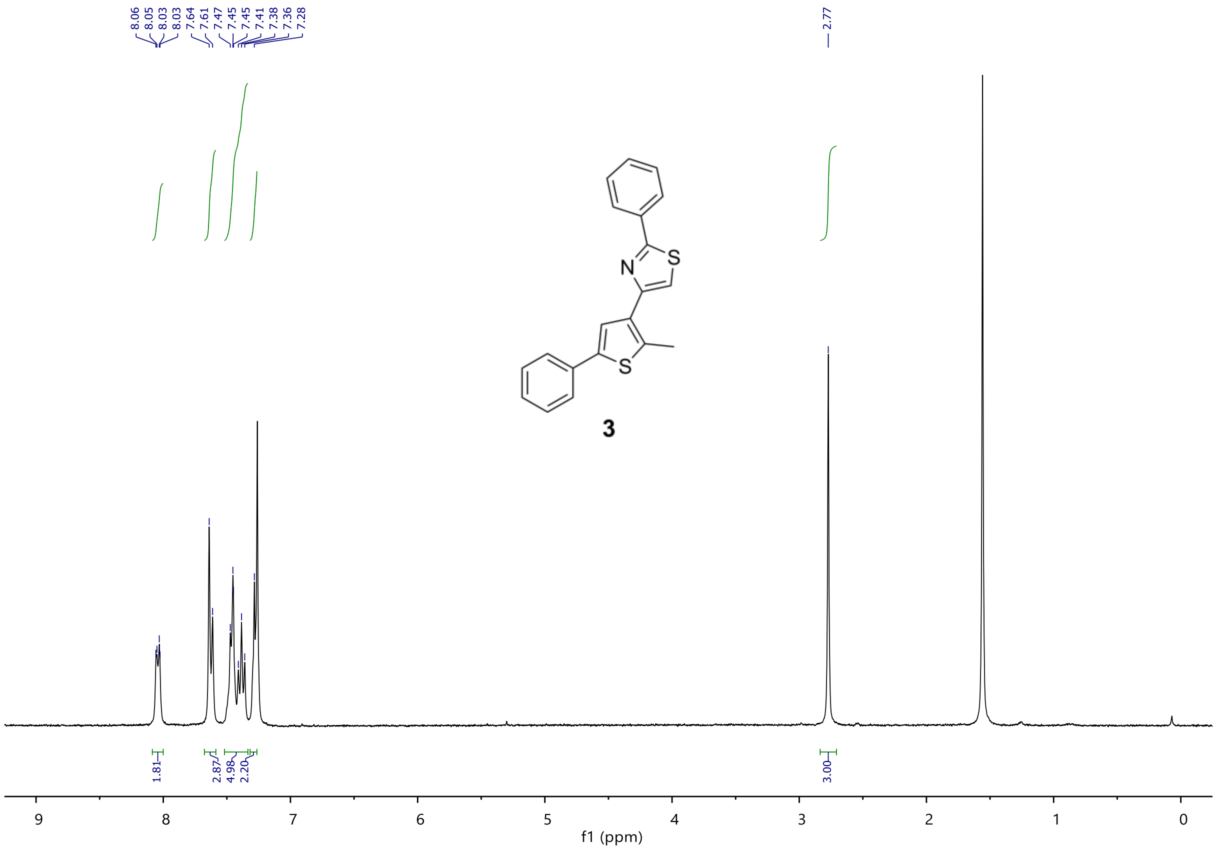

(c) Terarylene **1**

Adapting a published direct arylation procedure [6] **1** was synthesized as follows.

Xylene (mixture of isomers, 4 mL) was added to a Schlenk tube containing **3** (233 mg, 0.70 mmol), 2- methyl-3-bromo-5-(4-pyridyl)thiophene (179 mg, 0.70 mmol), pivalic acid (22 mg, 0.21 mmol), *t*- $Bu_2PMe.HBF_4$ (18 mg, 0.07 mmol), $Pd(OAc)_2$ (15 mg, 0.07 mmol) and Cs_2CO_3 (460 mg, 1.4 mmol) and the mixture was refluxed overnight. After addition of dichloromethane (10 mL) the solids in the mixture were removed by vacuum filtration and solids were washed with dichloromethane (2×10 mL). After removal of solvents the residue was purified by silica gel column chromatography (0 to 25% $EtOAc/CH_2Cl_2$) and the title compound was obtained as an amorphous pale yellow-greenish solid (175 mg, 49 % yield).

^{1}H-NMR (400 MHz, $CDCl_3$): δ (ppm) 8.57 (d, 2H), 8.06-8.03 (m, 2H), 7.51-7.46 (m, 5H), 7.38-7.31 (m, 5H), 7.25-7.21 (m, 2H), 2.35 (s, 3H), 2.26 (s, 3H).
^{13}C-NMR (101 MHz, $CDCl_3$): δ (ppm) 166.15, 150.45, 148.81, 140.92, 140.34, 140.06, 137.89, 137.49, 134.30, 133.53, 132.89, 130.32, 129.96, 129.09, 128.93, 127.60, 127.32, 127.09, 126.51, 125.50, 124.62, 119.50, 14.74, 14.63.
HRMS (ESI): calcd for $C_{30}H_{22}N_2S_3.H^+$: 507.1018 $[M+H]^+$, found: 507.1006.

^{1}H-NMR of **1**

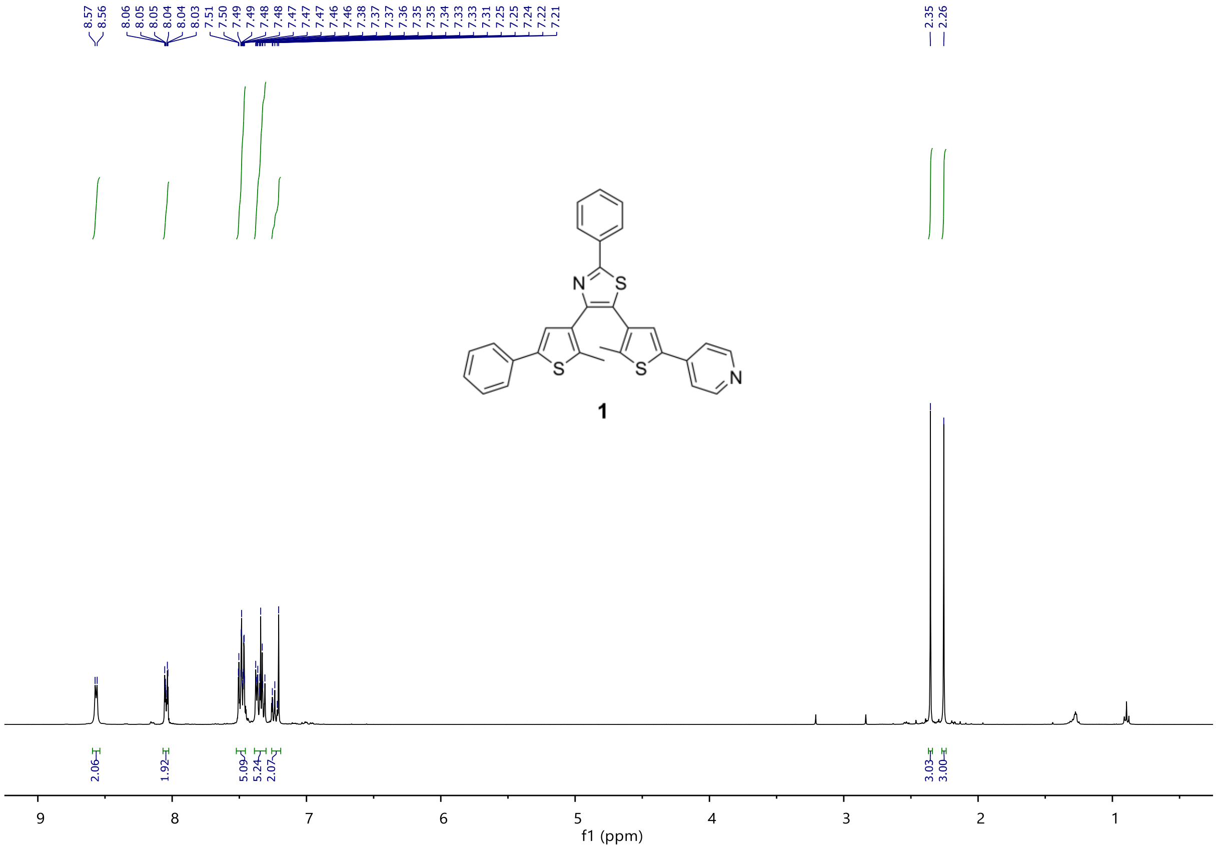

^{13}C-NMR of **1**

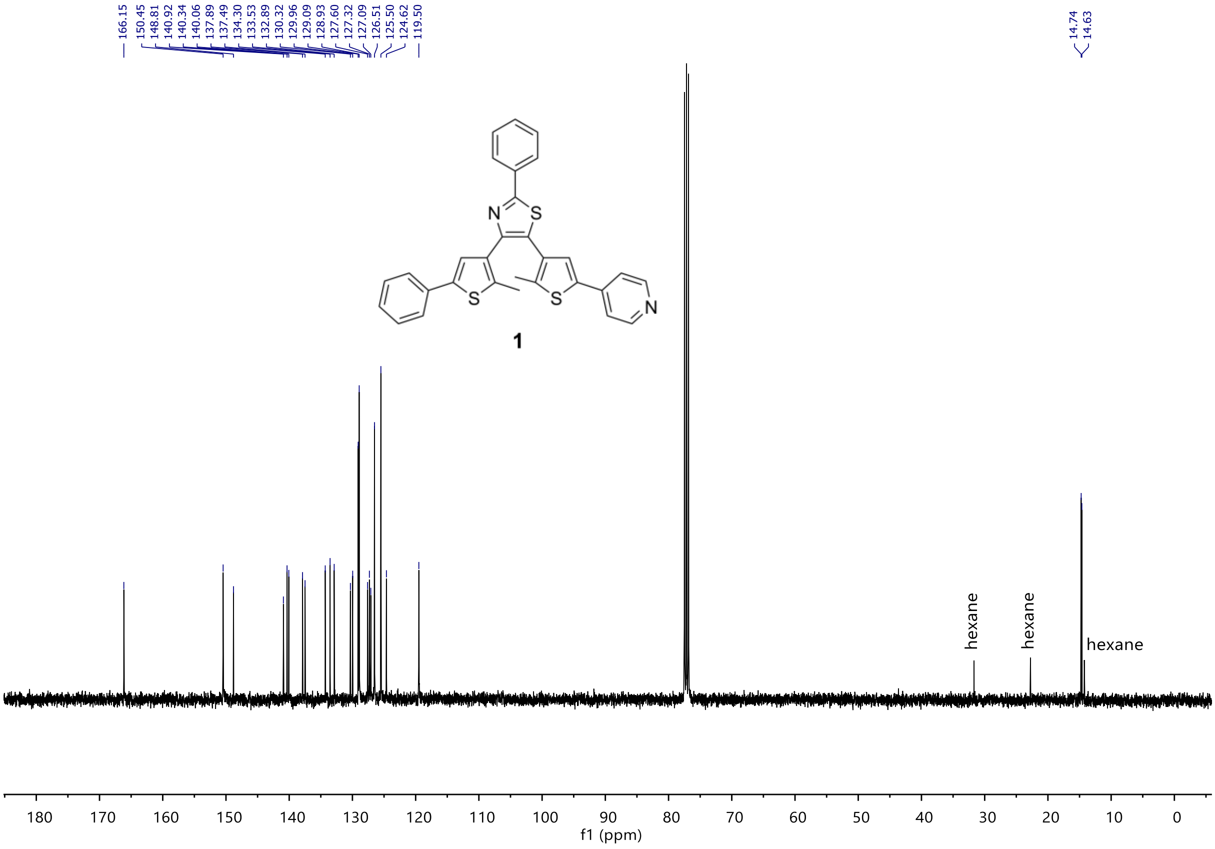

1.3 Synthetic protocols for **2**

(a) Compound **5**

5 was prepared in the same way as **4**.

To a solution of 2-methyl-3-bromo-5-(4-pyridyl)thiophene **8** (510 mg, 2 mmol) in dry diethyl ether (c.a. 30 mL) at -78°C (partial reprecipitation) was added dropwise *n*-BuLi (1 mL, 2.5 M in hexane). The resulting mixture was kept at that temperature for c.a. 2h 30 min. before neat 2- isopropoxy- 4,4,5,5- tetramethyl-1,3,2-dioxaborolane (0.5 mL, 2.5 mmol) was added quickly. The mixture was allowed to slowly warm back to RT overnight before aqueous NH_4Cl solution (20 mL, 1 M) was added. The aqueous phase was extracted twice with diethyl ether (20 mL each). The combined organic phase was washed with brine (20 mL), dried over Na_2SO_4 and filtered. After removal of solvents the residue solidified on standing in air and dried under vacuum to give **5** as a weakly colored solid (610 mg, quantitative). The crude **5** was used in the next step without further purification.
^{1}H-NMR (250 MHz, $CDCl_3$): δ (ppm) 8.52 (d, 2H), 7.70 (s, 1H), 7.50 (d, 2H), 2.73 (s, 3H), 1.34 (s, 12H).

^{1}H-NMR of **5**

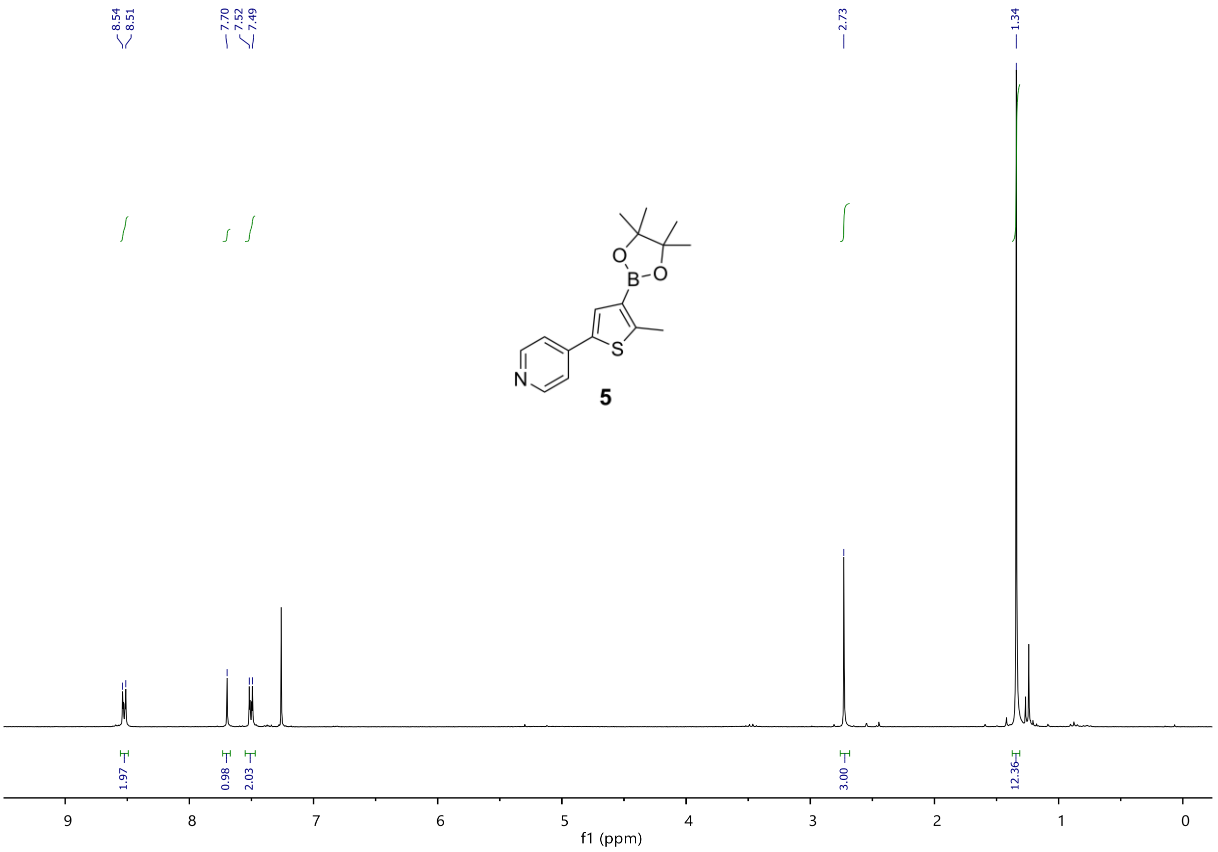

(b) Terarylene **2**

Anhydrous dioxane (25 mL) was added to a flask containing 2-Ph-4,5-dibromo-thiazole **6** (240 mg, 0.75 mmol), crude **5** (605 mg, 2 mmol), CsF (608 mg, 4 mmol) and $Pd(PPh_3)_4$ (60 mg, 0.05 mmol) and the resulting mixture was refluxed overnight. Dichloromethane and water (c.a. 20 mL of each) were added to the dark mixture and the aqueous phase was extracted with dichloromethane (3×20 mL). The combined organic phase was then washed with brine (c.a. 30 ml), dried over Na_2SO_4 and filtered. After removal of solvents the residue was purified by silica gel column chromatography (EtOAc to 5% EtOH/EtOAc) to give terarylene **2** as a straw yellow crystalline powder (240 mg, 63 % yield).

^{1}H-NMR (300 MHz, $CDCl_3$): δ (ppm) 8.58 (d, 2H), 8.53 (d, 2H), 8.04-8.01 (m, 2H), 7.49-7.47 (m, 3H), 7.40- 7.33 (m, 6H), 2.37 (s, 3H), 2.25 (s, 3H).

^{13}C-NMR (91 MHz, $CDCl_3$): δ (ppm) 166.48, 150.49, 150.34, 148.14, 141.23, 140.78, 140.53, 140.08, 137.78, 137.03, 133.53, 133.39, 130.45, 129.75, 129.13, 127.48, 127.43, 127.10, 126.50, 119.47, 119.44, 14.96, 14.59.

HRMS (ESI): calcd for $C_{29}H_{21}N_3S_3.H^+$: 508.0970 $[M+H]^+$, found: 508.0954.

^{1}H-NMR of **2**

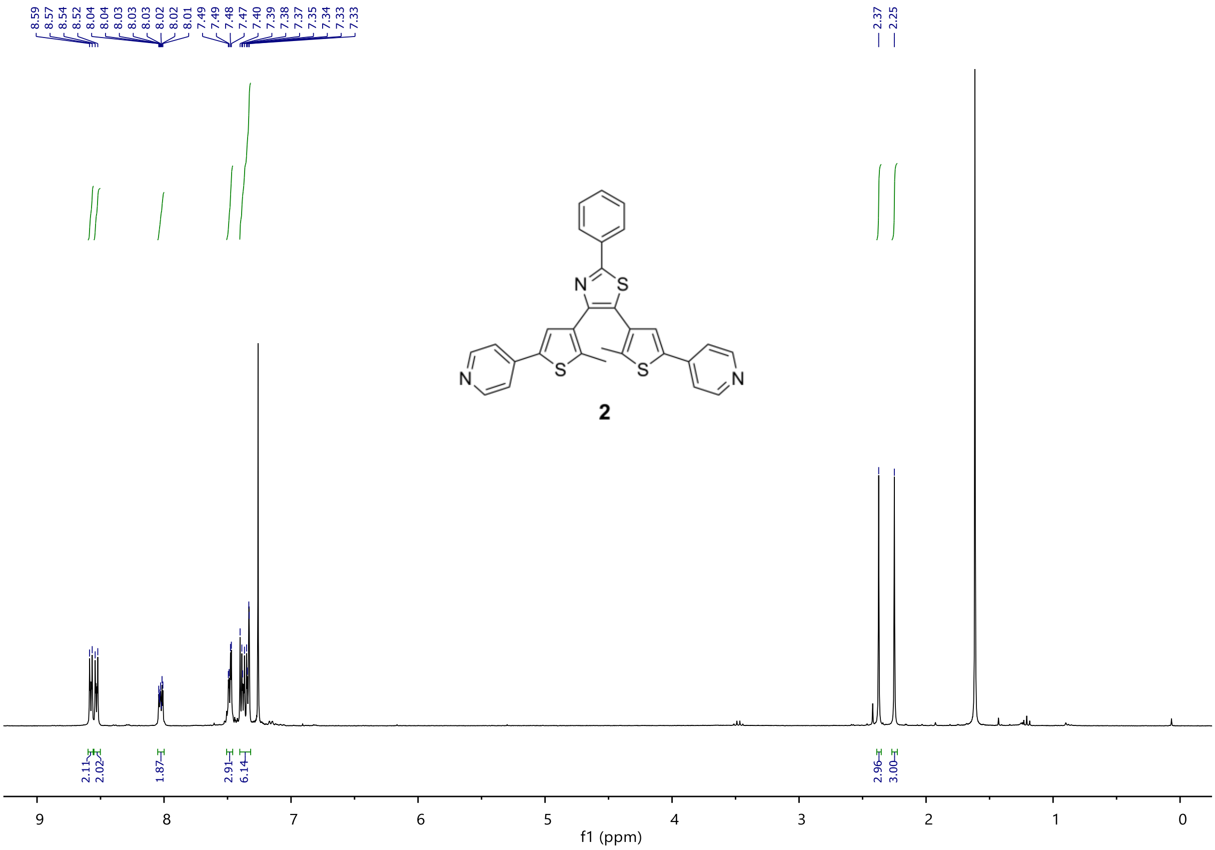

^{13}C-NMR of **2**

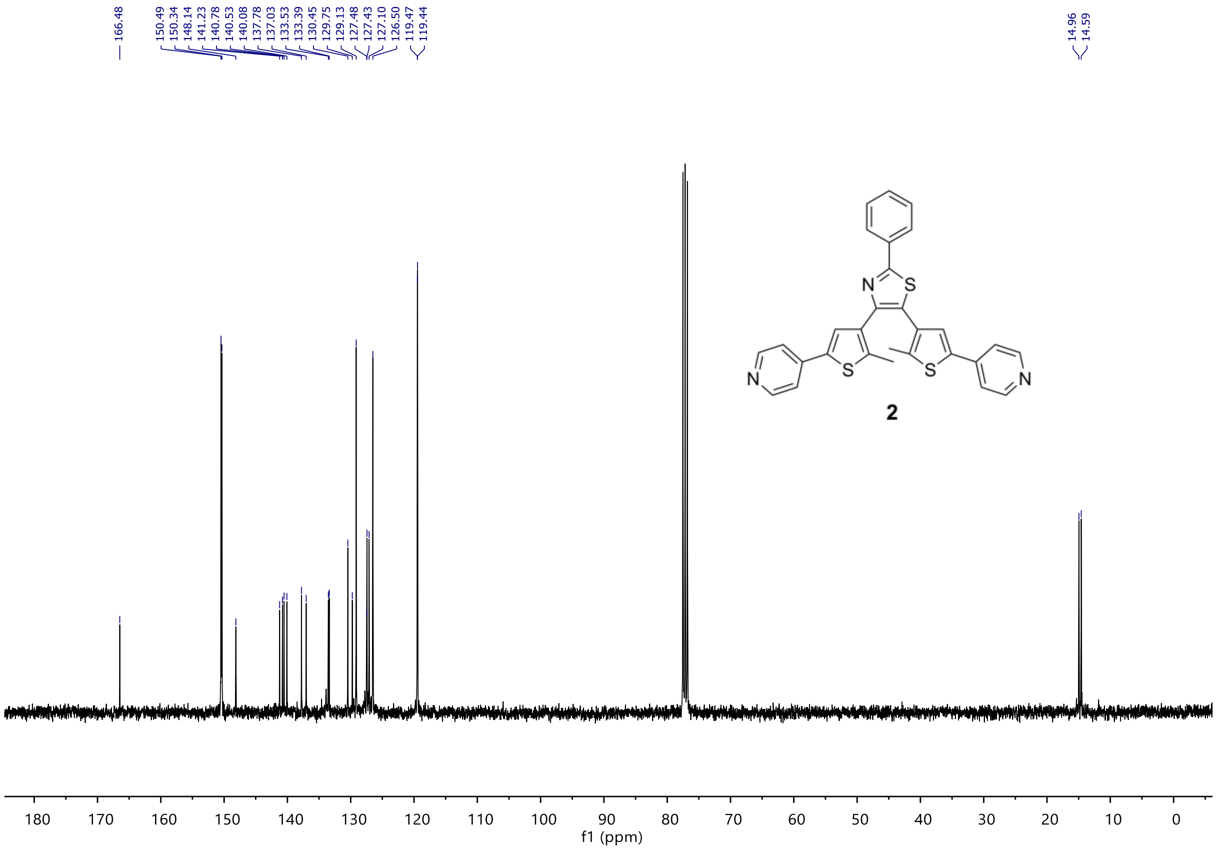

2. Spectroscopy and photokinetic experiments

2.1 General details

UV-vis absorption spectra were recorded on a Cary 5000 spectrophotometer from Agilent Technologies.

Photokinetic time-profiles were recorded using a home-made spectroscopy set-up, which collects absorption spectra at high rates under continuous irradiation of a solution sample, placed in a 1×1 cm quartz cuvette under strong stirring, with thermostat control at 20°C (sample holder LUMA40 from Quantum Northwest). A Hg-Xe lamp from Hamamatsu (Lightningcure LC8, 200 W), equipped with appropriate interferential filters (Semrock) to precisely select the desired wavelength at 313 nm Hg-lines, was used to photoisomerize the sample. Irradiation at 640 nm was delivered using a diode laser from Coherent (Obis LX 640). The incident irradiation power was measured by means of an Ophir PD300-UV photodiode, subtracting the NIR contribution from the total. A UV-visible Xenon lamp (75 W) was employed as the probe beam. The transmitted light through the sample was collected by a spectrograph equipped with a CCD camera (Princeton Instruments). The absorption time-profiles at the band maximum of the DAE in the closed form were plotted and fitted using a numerical iterative fitting method implemented in the Igor Pro software (Wavemetrics), providing the $\Phi_{OF\rightarrow CF}$ and $\Phi_{CF\rightarrow OF}$ quantum yields.

2.2 Photokinetic profiles

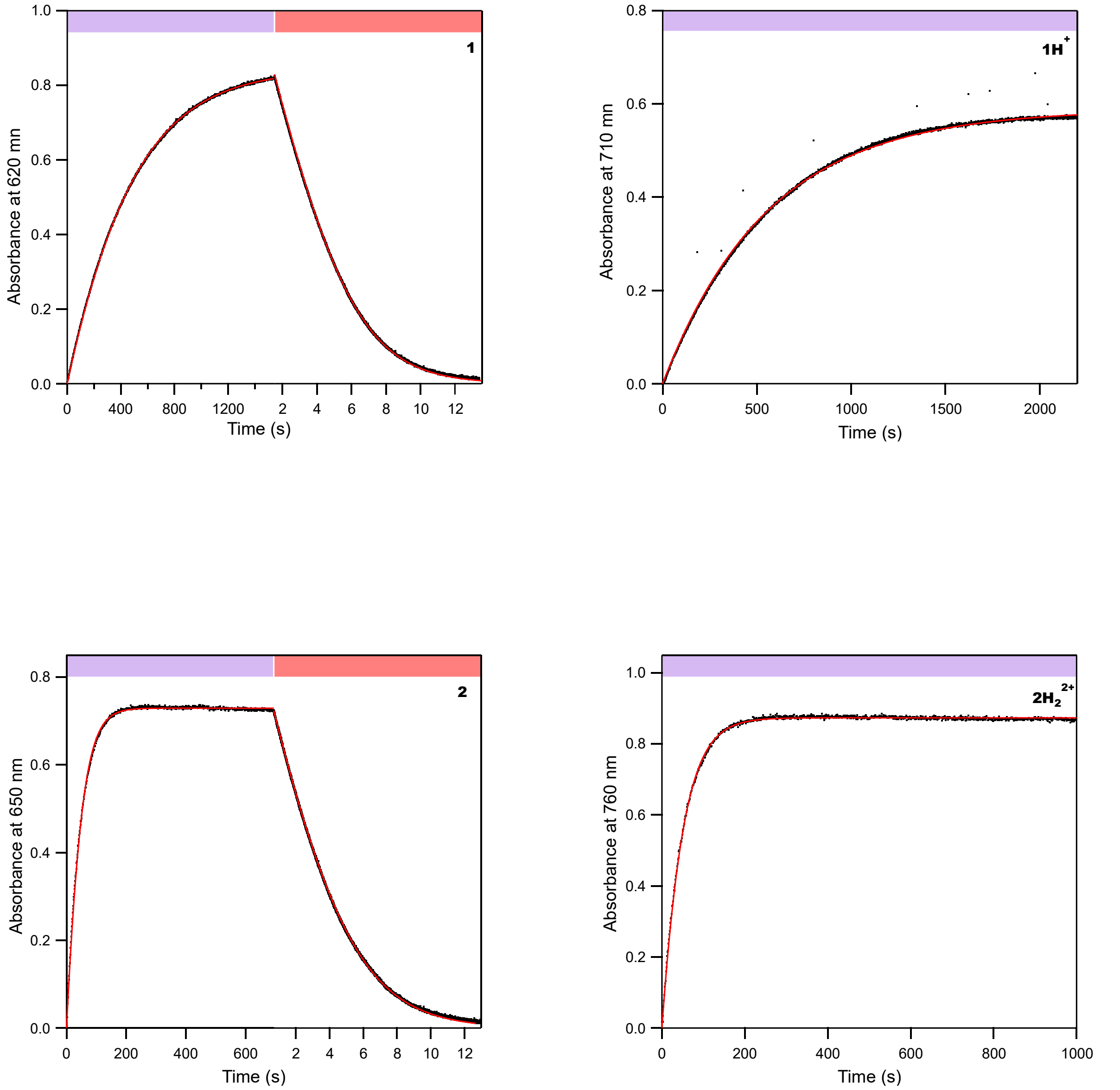

Figure S2: Photokinetics of photochromic reactions under illumination at 313 nm (purple stripe) or 640 nm (red stripe) for compounds **1** and **2** in acetonitrile in absence and presence of 10 equivalents of PTSA. Curve fitting are added in full red lines.

3. DFT calculations

The geometry optimizations were obtained using the CAM Becke-3-Lee-Yang-Parr (CAM-B3LYP) hybrid exchange-correlation functional[7] with the 6-31G+(d,p) basis set in acetonitrile (conductor-like polarizable continuum model, CPCM), as implemented in Gaussian 16 software package. Absence of negative frequencies was checked to ensure true minimum determination. The first Franck-Condon energy transitions and corresponding transitions moments were calculated by the time-dependent DFT formalism (TD-DFT) with the same functional and basis set.

The optimised geometries and the calculated electronic transitions are displayed in Figure S3.

a)

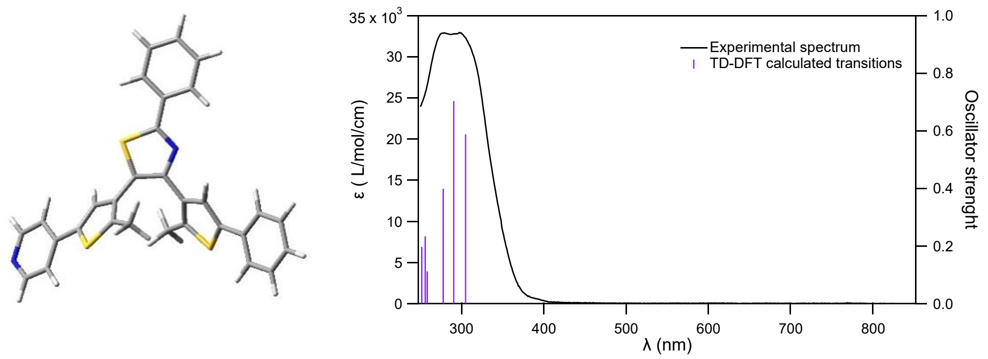

35 x 10³
ε (L/mol/cm)
λ (nm)
Oscillator strenght
Experimental spectrum
TD-DFT calculated transitions

b)

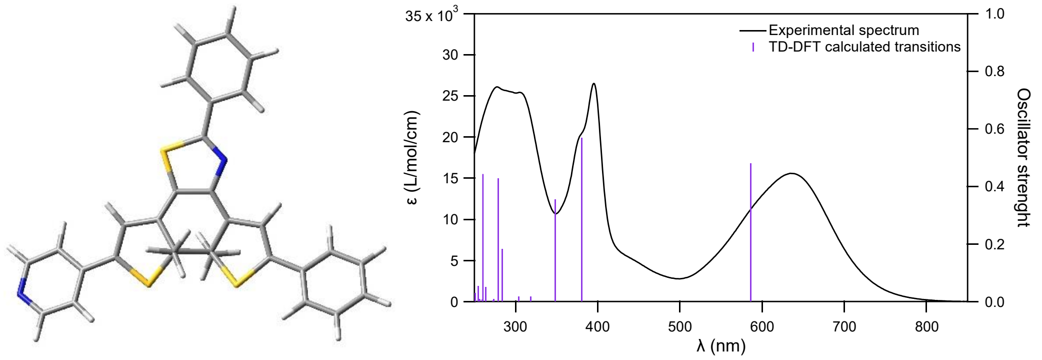

35 x 10³
ε (L/mol/cm)
λ (nm)
Oscillator strenght
Experimental spectrum
TD-DFT calculated transitions

c)

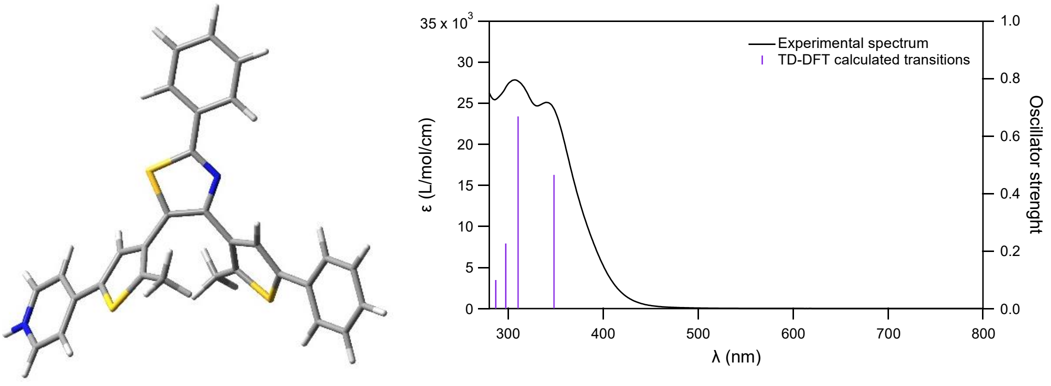

35 x 10³
ε (L/mol/cm)
λ (nm)
Oscillator strenght
Experimental spectrum
TD-DFT calculated transitions

d)

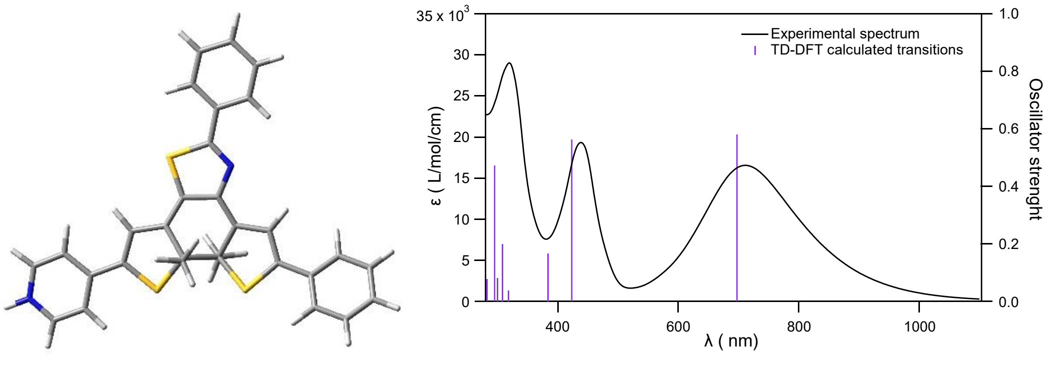

35 x 10³
ε (L/mol/cm)
λ (nm)
Oscillator strenght
Experimental spectrum
TD-DFT calculated transitions

e)

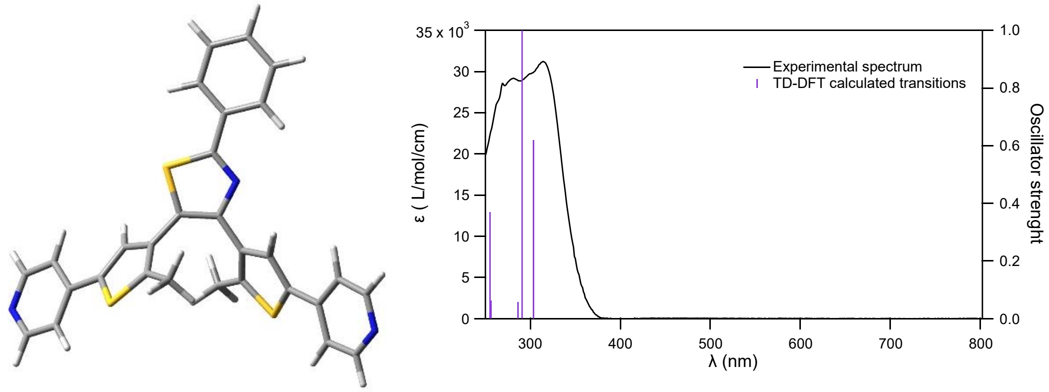

f)

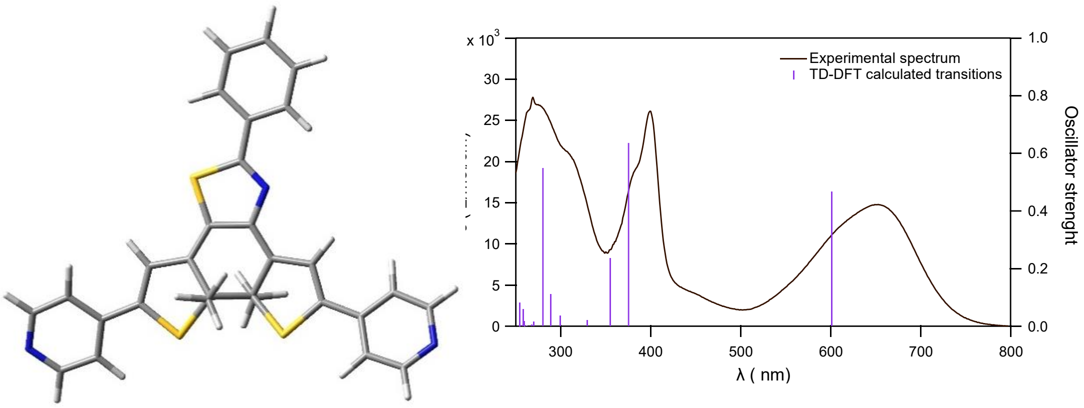

g)

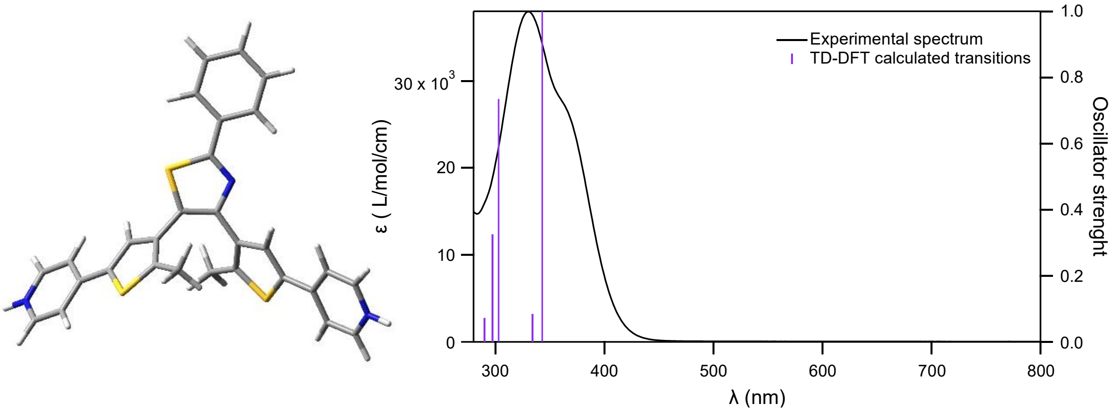

h)

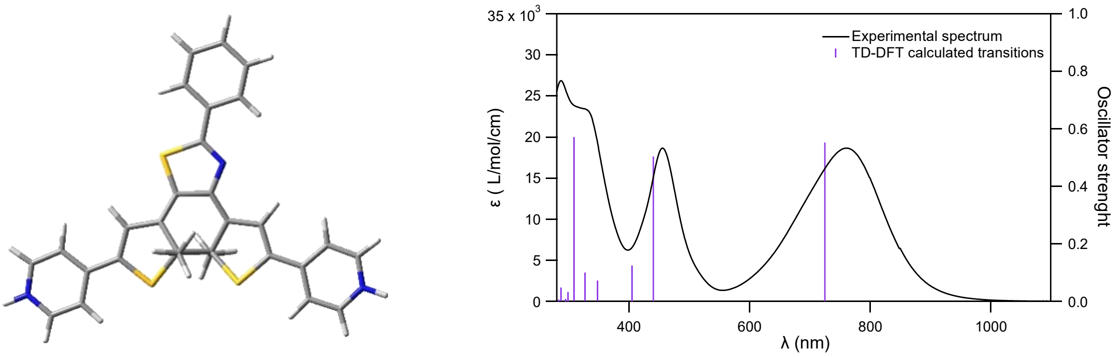

Figure S3: Optimised geometry structures and TD-DFT calculated in CPCM acetonitrile for a) **1**(OF) b) **1**(CF) c) **1**·H$^+$(OF) d) **1**·H$^+$(CF) e) **2**(OF) f) **2**(CF) g) **2**·H_2^{2+}(OF) h) **2**·H_2^{2+}(CF).

Frontier molecular orbitals of the open forms are displayed in Figure S4.

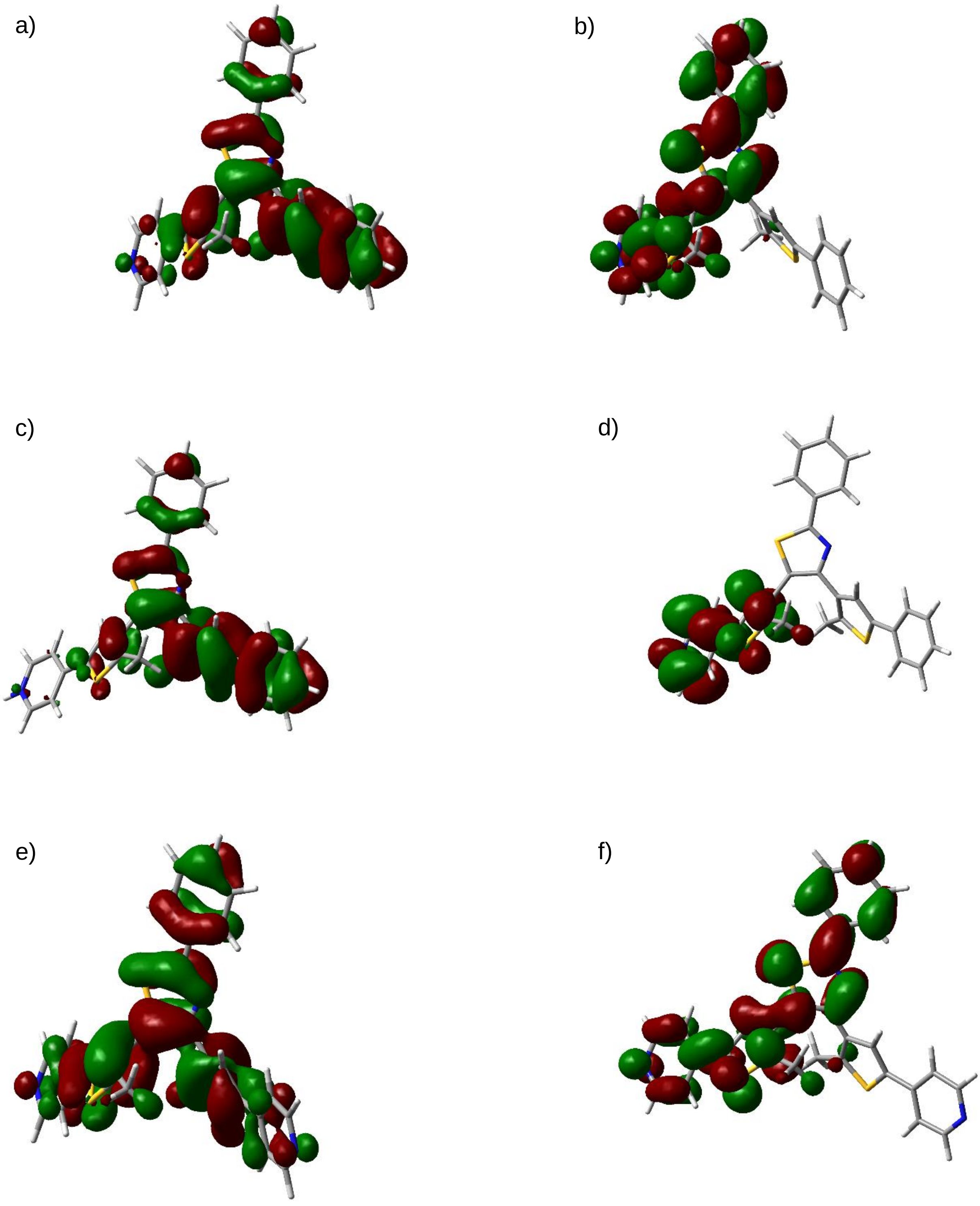

g)

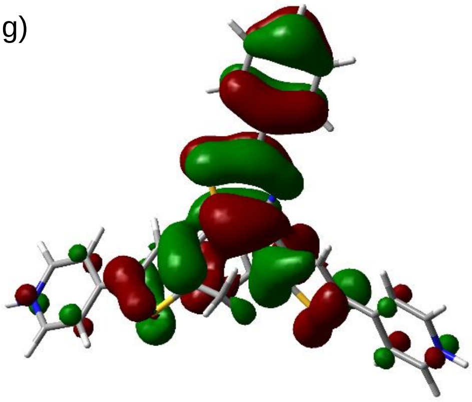

h)

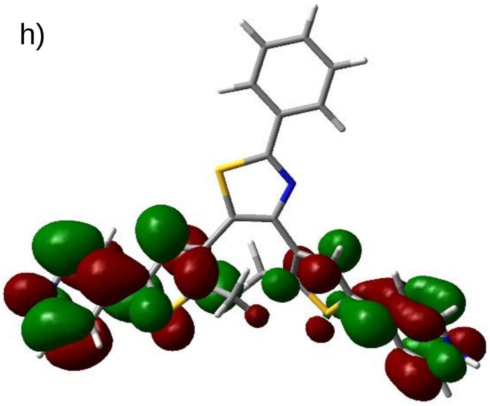

Figure S4: Calculated molecular orbitals of the OF for **1** a) HOMO b) LUMO ; **1**·H^+ c) HOMO d) LUMO ; **2** e) HOMO f) LUMO ; **2**·H_2^{2+} g) HOMO h) LUMO.

Table S1: Values of $\Delta G_{c\text{-}o}$ calculated from the difference in the computationally calculated "Sum of Electronic and Thermal Free Energies" of the open and closed forms of each pair of photochromic isomers.

	$\Delta G_{c\text{-}o}$ [kJ·mol⁻¹]
**1**	99
**1**·H^+	102
**2**	103
**2**·H_2^{2+}	118

4. Activation energy determination

The activation parameters, based on Arrhenius equation, of both compounds in neutral and protonated state were determined by performing thermal back reaction in acetonitrile at various temperature ranging from 10°C to 60°C, thus providing the kinetic constant at these different temperatures. Arrhenius plots are shown in Figure S5.

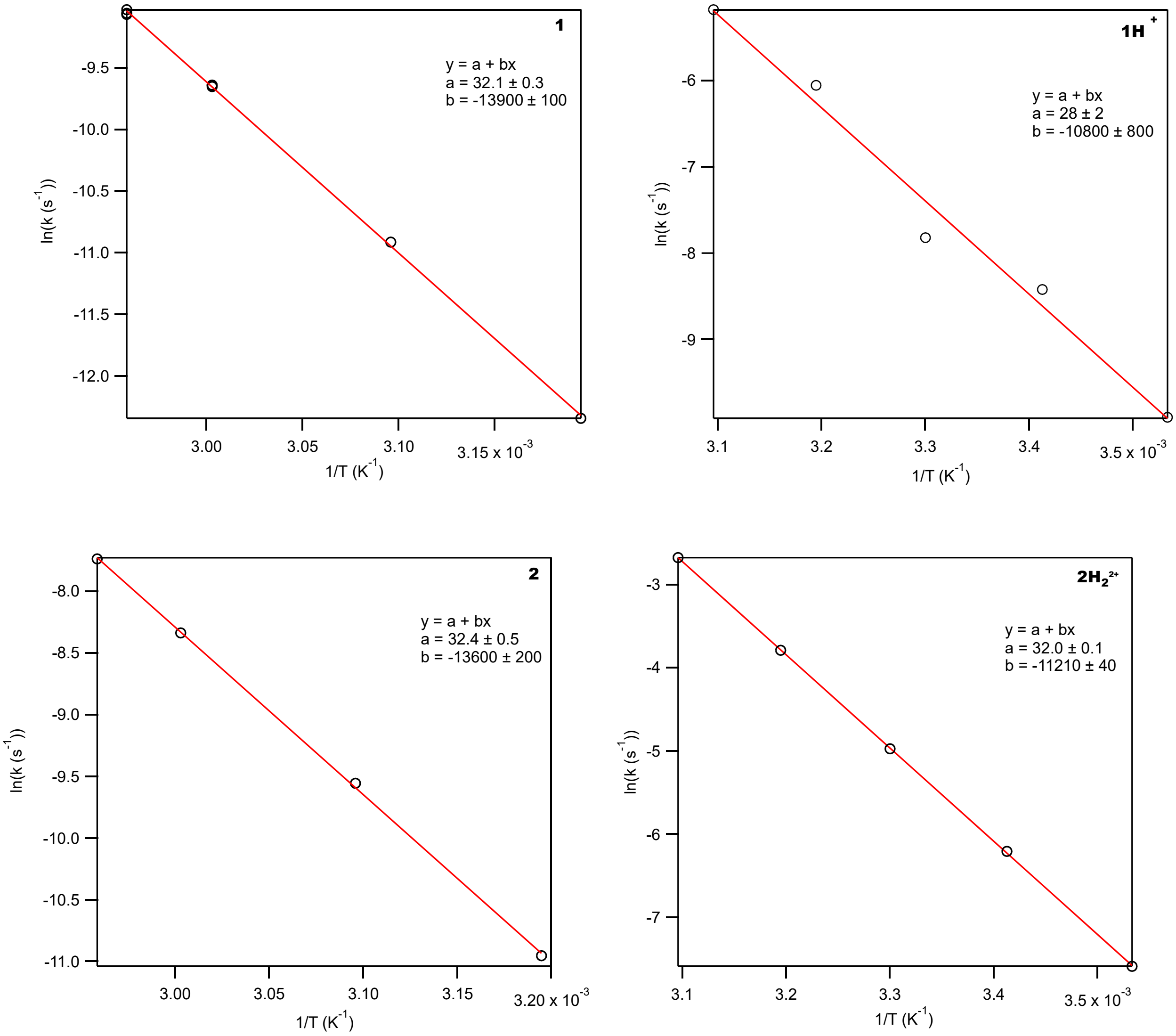

Figure S5: Arrhenius plots for compounds **1** and **2** in acetonitrile in absence and presence of 10 equivalents of PTSA.

5. Acidic titrations

The titration of the open form of both molecules was performed by UV-visible spectroscopy, by progressive addition of a 1.2 10^{-2} M solution of PTSA in acetonitrile to a solution of **1** (resp. **2**) at 9.5 10^{-5} M (resp. 4.5 10^{-5} M).

The titration of the closed form of both molecules was performed by UV-visible spectroscopy, by independent addition of a 1.2 10^{-2} M solution of PTSA in acetonitrile to a solution of **1** (resp. **2**) at 4.0 10^{-5} M (resp. 4.7 10^{-5} M).

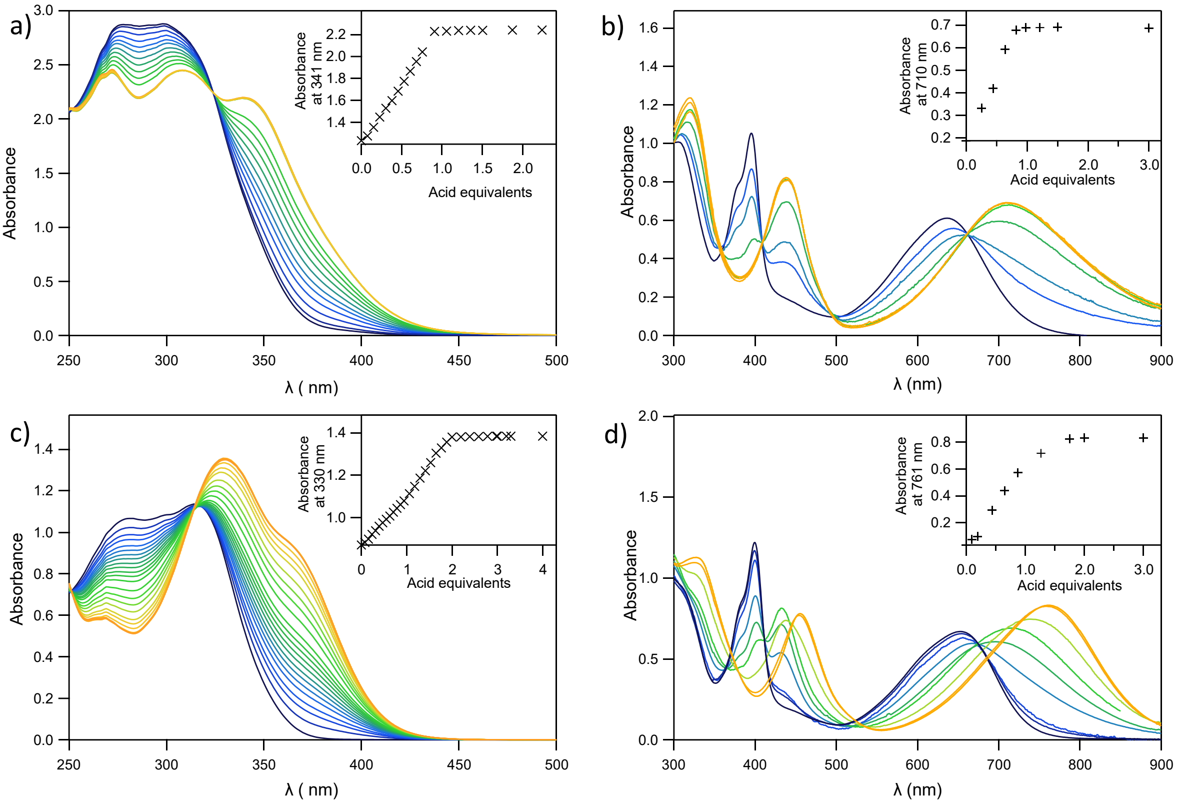

Figure S6: UV-visible spectral change (blue to green to yellow solid lines) during the acidic titration of a) **1**(OF) b) **1**(CF) c) **2**(OF) and d) **2**(CF). The evolution of absorbance characteristics of the protonated forms is displayed in inset.

6. Catalyzed back reaction

6.1 General procedure for acid catalyzed back reaction.

A solution of **1** (resp. **2**) of 4.03 10^{-5} M (resp. 4.71 10^{-5} M) was irradiated at 313 nm until photostationary state was reached. The appropriate volume of a solution of PTSA at 1.14 10^{-2} M was then quickly added and UV-visible spectra were recorded at a rate varying from 1 spectrum per 2 minutes to 1 spectrum per 15 minutes depending on the experiment.

6.2 Kinetic equations

The fitting of absorbance decay in presence of catalytic amounts of acid was done on Python™, based on the following kinetic equations.

$$\frac{d[\mathrm{OF}]}{dt} = \mathrm{k_0[CF]} - \mathrm{k_{O1}[OF][H^+]} + \mathrm{k_{O-1}\,[OFH^+]}$$

$$\frac{d[\mathrm{OFH^+}]}{dt} = \mathrm{k_1[CFH^+]} + \mathrm{k_{O1}[OF][H^+]} - \mathrm{k_{O-1}\,[OFH^+]} - \mathrm{k_{O1}[OFH^+][H^+]} + \mathrm{k_{O-1}\,[OFH_2^{2+}]}$$

$$\frac{d[\mathrm{OFH_2}^{2+}]}{dt} = \mathrm{k_2[CFH_2^{2+}]} + \mathrm{k_{O1}[OFH^+][H^+]} - \mathrm{k_{O-1}\,[OFH_2^{2+}]}$$

$$\frac{d[\mathrm{CF}]}{dt} = -\mathrm{k}_0[\mathrm{CF}] - \mathrm{k}_{\mathrm{C1}}[\mathrm{CF}][\mathrm{H}^+] + \mathrm{k}_{\mathrm{C-1}}[\mathrm{CFH}^+]$$

$$\frac{d[\mathrm{CFH}^+]}{dt} = -\mathrm{k}_1[\mathrm{CFH}^+] + \mathrm{k}_{\mathrm{C1}}[\mathrm{CF}][\mathrm{H}^+] - \mathrm{k}_{\mathrm{C-1}}[\mathrm{CFH}^+] - \mathrm{k}_{\mathrm{C2}}[\mathrm{CFH}^+][\mathrm{H}^+] + \mathrm{k}_{\mathrm{C-2}}[\mathrm{CFH}_2^{2+}]$$

$$\frac{d[\mathrm{CFH_2}^{2+}]}{dt} = -\mathrm{k}_2[\mathrm{CFH}_2^{2+}] + \mathrm{k}_{\mathrm{C2}}[\mathrm{CFH}^+][\mathrm{H}^+] - \mathrm{k}_{\mathrm{C-2}}[\mathrm{CFH}_2^{2+}]$$

$$\frac{d[\mathrm{H}^+]}{dt} = -\mathrm{k}_{\mathrm{O1}}([\mathrm{OF}] + [\mathrm{OFH}^+])[\mathrm{H}^+] + \mathrm{k}_{\mathrm{O-1}}([\mathrm{OFH}^+] + [\mathrm{OFH}_2^{2+}]) - (\mathrm{k}_{\mathrm{C1}}[\mathrm{CF}] + \mathrm{k}_{\mathrm{C2}}[\mathrm{CFH}^+])[\mathrm{H}^+] + \mathrm{k}_{\mathrm{C-1}}[\mathrm{CFH}^+] + \mathrm{k}_{\mathrm{C-2}}[\mathrm{CFH}_2^{2+}]$$

6.3 Supplementary data for acid catalyzed back reaction

For each experiment, the following data provide the absorption spectra monitoring the back reaction associated to the recording rate, the simulated distribution curves of every species in solution and the simulated TON curves, as defined in main text, the dashed lines correspond to the maximum theoretical TON values, also defined in main text. Finally, individual fitting parameters for each experiment are indicated as well as the average values given in the main text, for comparison purposes.

*Compound **1** in presence of 0.1 equivalent of PTSA*

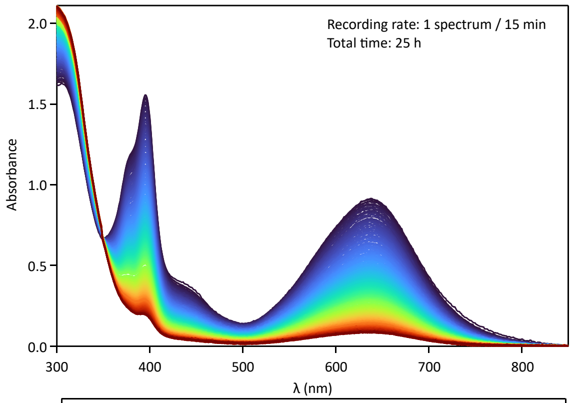

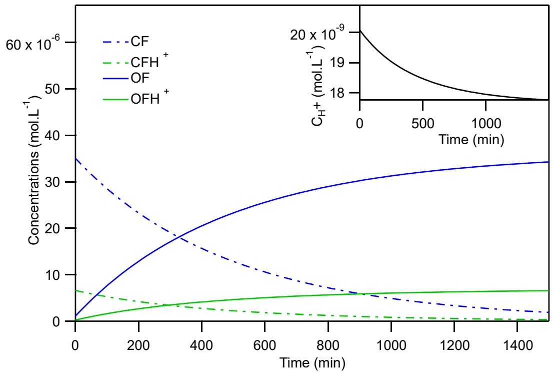

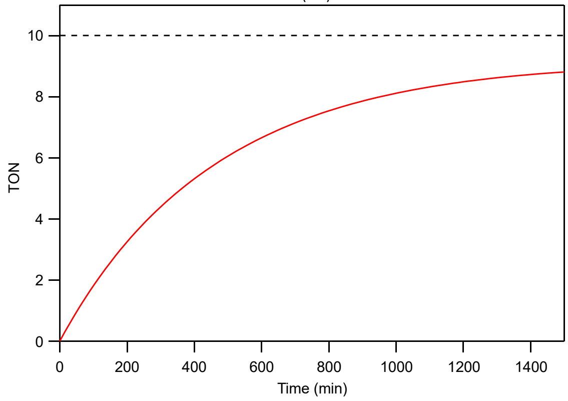

$k_{O1,ind}$ ($k_{O1,av}$) [×10^6 s^{-1}mol^{-1}L]	$k_{O-1,ind}$ ($k_{O-1,av}$) [×10^{-1} s^{-1}]	$k_{C1,ind}$ ($k_{C1,av}$) [×10^6 s^{-1}mol^{-1}L]	$k_{C-1,ind}$ ($k_{C-1,av}$) [×10^{-1} s^{-1}]
1.95 (1.7)	1.81 (1.2)	1.89 (1.8)	2.00 (2.2)

*Compound **1** in presence of 0.3 equivalent of PTSA*

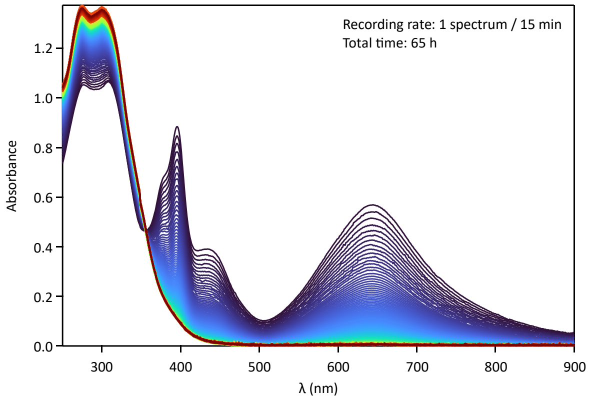

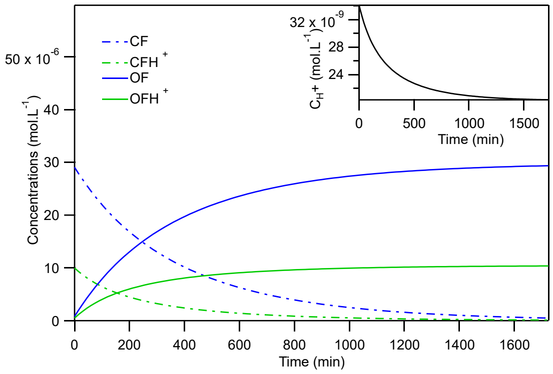

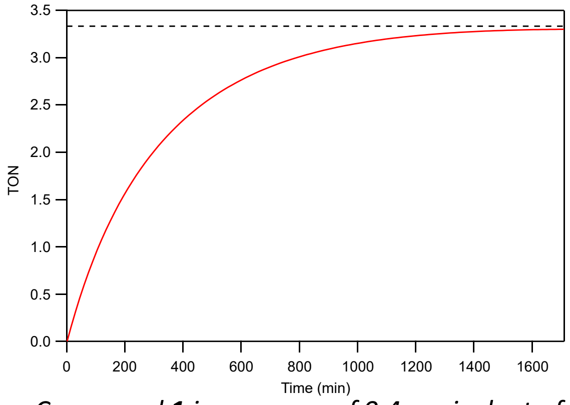

$k_{O1,ind}$ ($k_{O1,av}$) [×10^6 s^{-1}mol^{-1}L]	$k_{O-1,ind}$ ($k_{O-1,av}$) [×10^{-1} s^{-1}]	$k_{C1,ind}$ ($k_{C1,av}$) [×10^6 s^{-1}mol^{-1}L]	$k_{C-1,ind}$ ($k_{C-1,av}$) [×10^{-1} s^{-1}]
1.98 (1.7)	1.14 (1.2)	2.02 (1.8)	2.01 (2.2)

*Compound **1** in presence of 0.4 equivalent of PTSA*

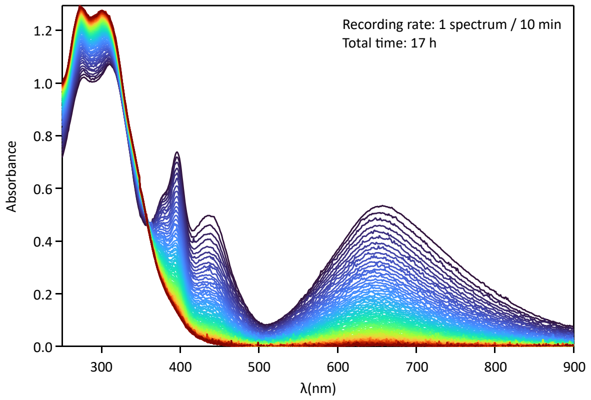

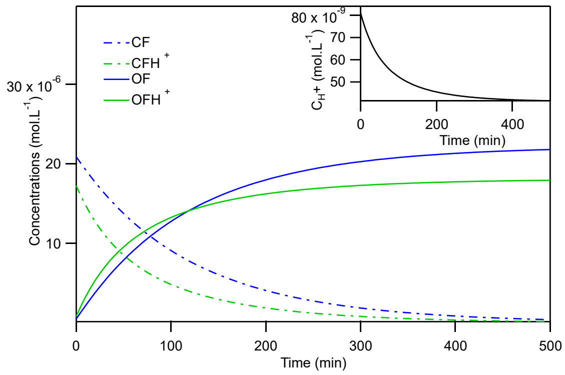

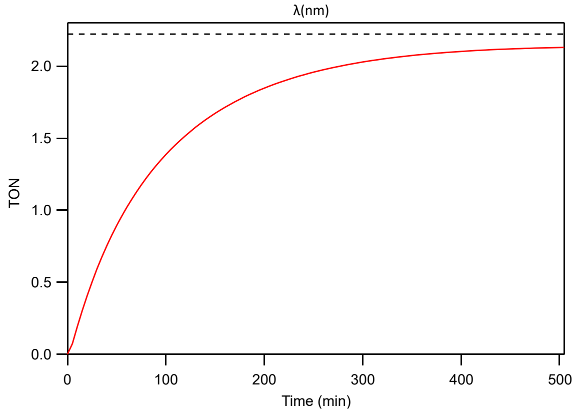

$k_{O1,ind}$ ($k_{O1,av}$) [×10^6 s^{-1}mol^{-1}L]	$k_{O-1,ind}$ ($k_{O-1,av}$) [×10^{-1} s^{-1}]	$k_{C1,ind}$ ($k_{C1,av}$) [×10^6 s^{-1}mol^{-1}L]	$k_{C-1,ind}$ ($k_{C-1,av}$) [×10^{-1} s^{-1}]
2.02 (1.7)	1.02 (1.2)	1.97 (1.8)	2.02 (2.2)

*Compound **1** in presence of 0.7 equivalent of PTSA*

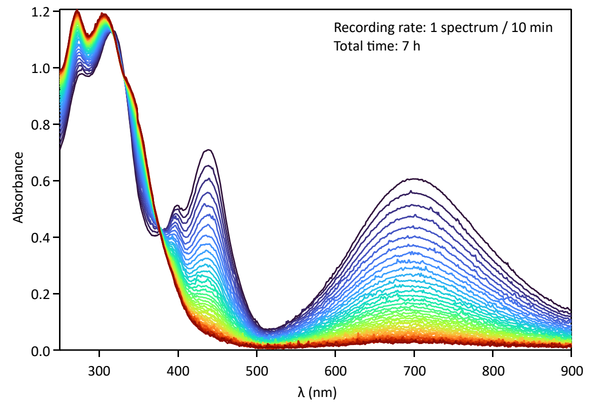

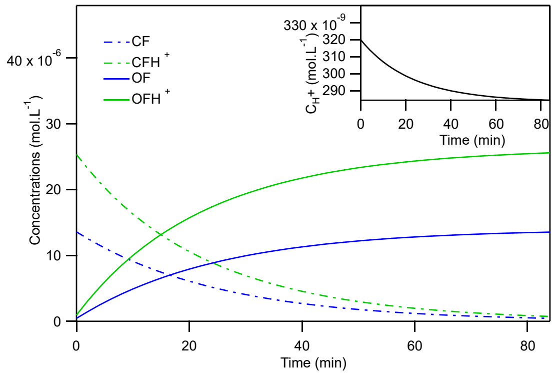

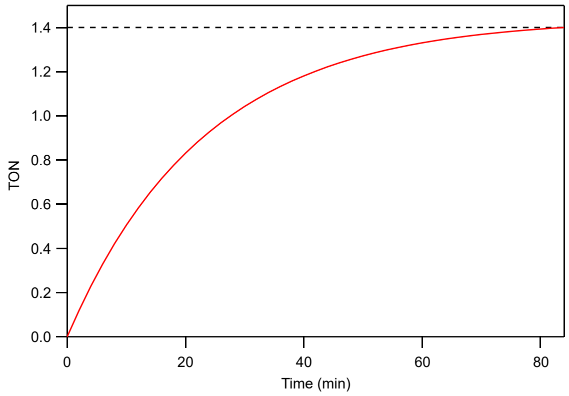

$k_{O1,ind}$ ($k_{O1,av}$) [$\times10^{6}$ $s^{-1}mol^{-1}L$]	$k_{O-1,ind}$ ($k_{O-1,av}$) [$\times10^{-1}$ s^{-1}]	$k_{C1,ind}$ ($k_{C1,av}$) [$\times10^{6}$ $s^{-1}mol^{-1}L$]	$k_{C-1,ind}$ ($k_{C-1,av}$) [$\times10^{-1}$ s^{-1}]
1.03 (1.7)	1.55 (1.2)	1.48 (1.8)	2.54 (2.2)

*Compound **2** in presence of 0.1 equivalent of PTSA*

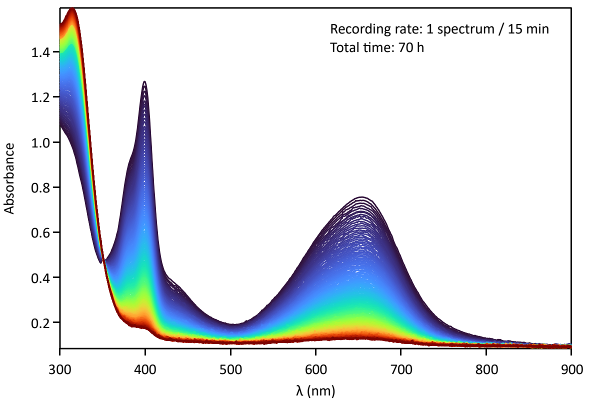

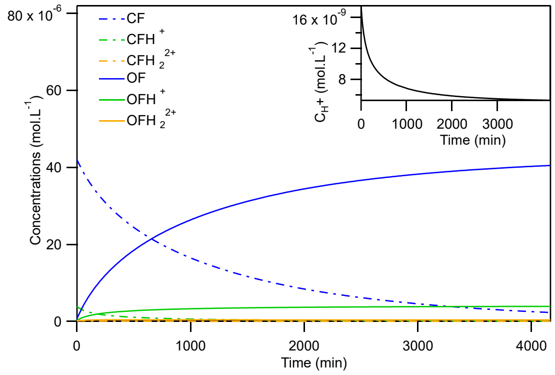

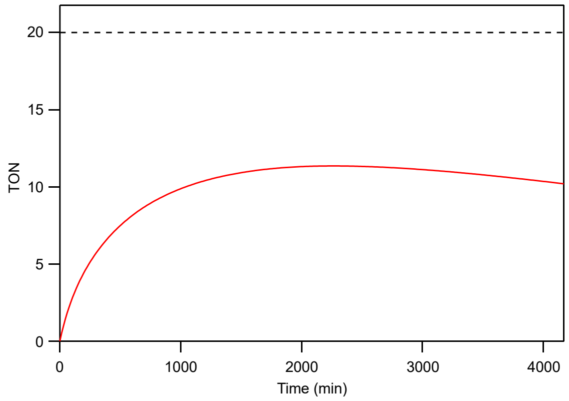

$k_{O1,ind}$ ($k_{O1,av}$) [$\times10^{6}$ $s^{-1}mol^{-1}L$]	$k_{O-1,ind}$ ($k_{O-1,av}$) [$\times10^{-1}$ s^{-1}]	$k_{C1,ind}$ ($k_{C1,av}$) [$\times10^{6}$ $s^{-1}mol^{-1}L$]	$k_{C-1,ind}$ ($k_{C-1,av}$) [$\times10^{-1}$ s^{-1}]	$k_{C2,ind}$ ($k_{C2,av}$) [$\times10^{6}$ $s^{-1}mol^{-1}L$]	$k_{C-2,ind}$ ($k_{C-2,av}$) [$\times10^{-1}$ s^{-1}]	$k_{1,ind}$ ($k_{1,av}$) [$\times10^{-4}$ s^{-1}]
1.92 (1.8)	1.06 (1.2)	1.65 (1.8)	3.11 (2.2)	1.95 (1.9)	0.59 (0.59)	2.30 (2.0)

*Compound **2** in presence of 0.2 equivalent of PTSA*

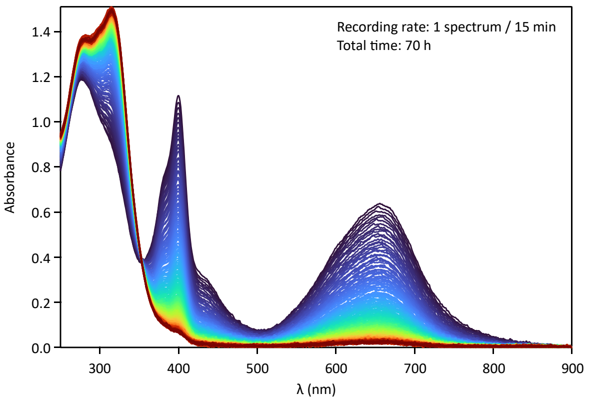

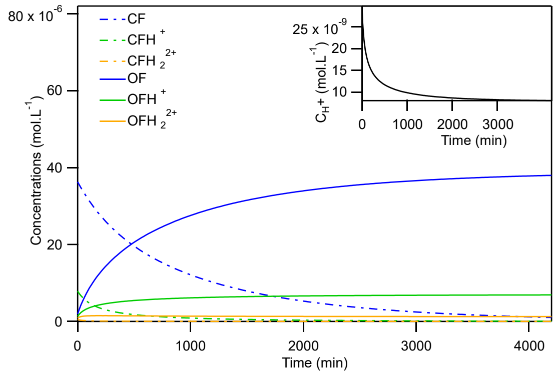

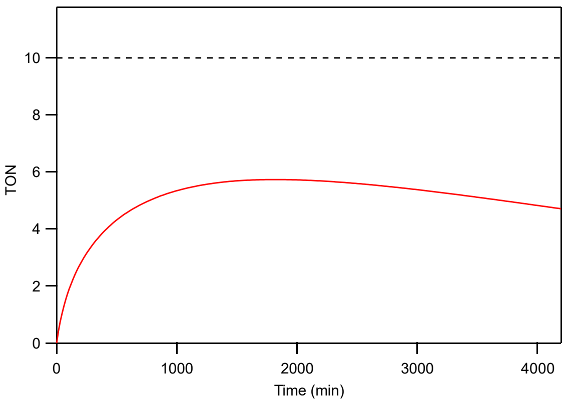

$k_{O1,ind}$ ($k_{O1,av}$) [$\times 10^6$ $s^{-1}mol^{-1}L$]	$k_{O-1,ind}$ ($k_{O-1,av}$) [$\times 10^{-1}$ s^{-1}]	$k_{C1,ind}$ ($k_{C1,av}$) [$\times 10^6$ $s^{-1}mol^{-1}L$]	$k_{C-1,ind}$ ($k_{C-1,av}$) [$\times 10^{-1}$ s^{-1}]	$k_{C2,ind}$ ($k_{C2,av}$) [$\times 10^6$ $s^{-1}mol^{-1}L$]	$k_{C-2,ind}$ ($k_{C-2,av}$) [$\times 10^{-1}$ s^{-1}]	$k_{1,ind}$ ($k_{1,av}$) [$\times 10^{-4}$ s^{-1}]
1.95 (1.8)	0.86 (1.2)	1.95 (1.8)	4.09 (2.2)	1.82 (1.9)	0.53 (0.59)	1.95 (2.0)

*Compound **2** in presence of 0.4 equivalent of PTSA*

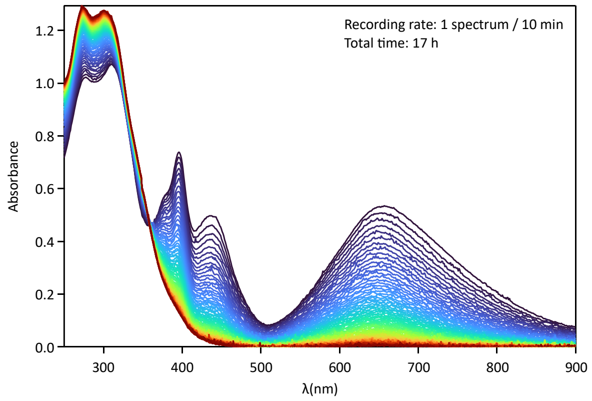

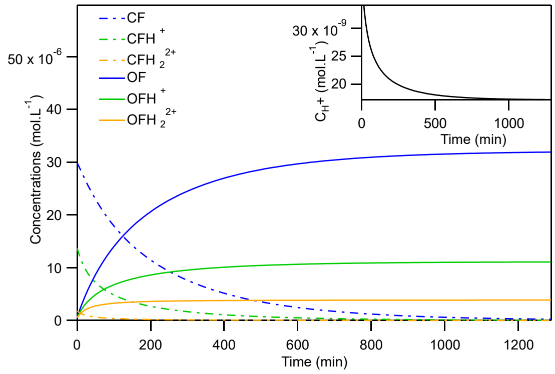

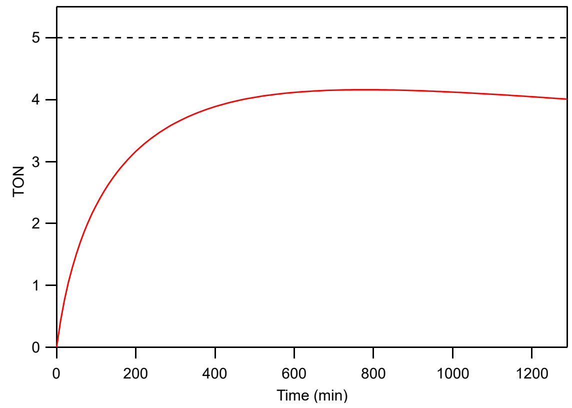

$k_{O1,ind}$ ($k_{O1,av}$) [$\times 10^6$ $s^{-1}mol^{-1}L$]	$k_{O-1,ind}$ ($k_{O-1,av}$) [$\times 10^{-1}$ s^{-1}]	$k_{C1,ind}$ ($k_{C1,av}$) [$\times 10^6$ $s^{-1}mol^{-1}L$]	$k_{C-1,ind}$ ($k_{C-1,av}$) [$\times 10^{-1}$ s^{-1}]	$k_{C2,ind}$ ($k_{C2,av}$) [$\times 10^6$ $s^{-1}mol^{-1}L$]	$k_{C-2,ind}$ ($k_{C-2,av}$) [$\times 10^{-1}$ s^{-1}]	$k_{1,ind}$ ($k_{1,av}$) [$\times 10^{-4}$ s^{-1}]
2.02 (1.8)	1.00 (1.2)	2.14 (1.8)	1.89 (2.2)	1.95 (1.9)	0.611 (0.59)	1.92 (2.0)

*Compound **2** in presence of 0.6 equivalent of PTSA*

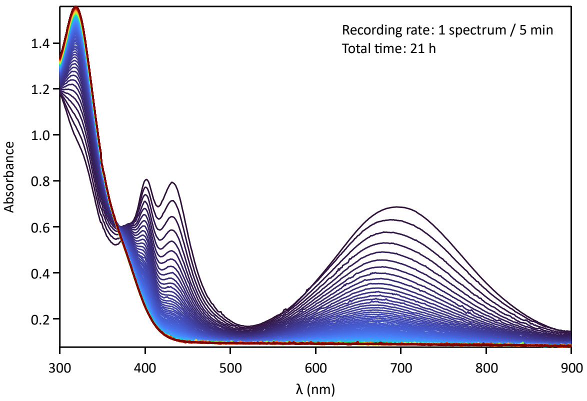

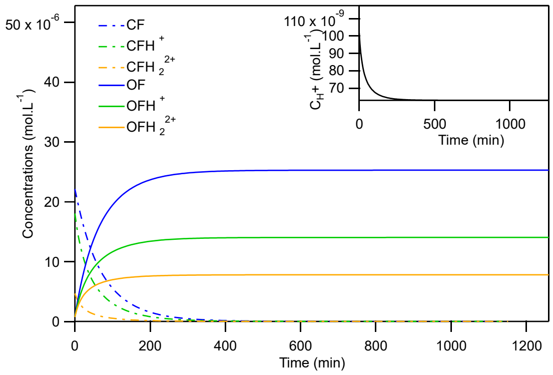

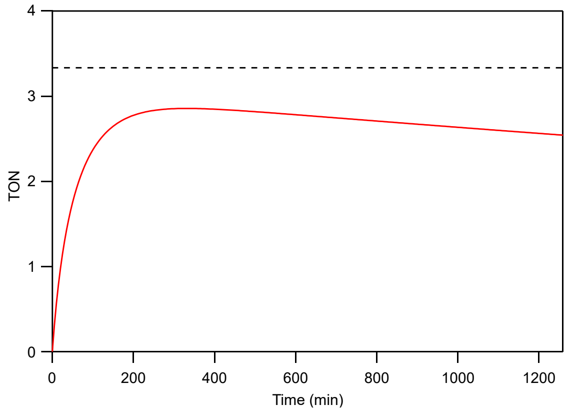

$k_{O1,ind}$ ($k_{O1,av}$) [×10^6 s^−1mol^−1L]	$k_{O-1,ind}$ ($k_{O-1,av}$) [×10^−1 s^−1]	$k_{C1,ind}$ ($k_{C1,av}$) [×10^6 s^−1mol^−1L]	$k_{C-1,ind}$ ($k_{C-1,av}$) [×10^−1 s^−1]	$k_{C2,ind}$ ($k_{C2,av}$) [×10^6 s^−1mol^−1L]	$k_{C-2,ind}$ ($k_{C-2,av}$) [×10^−1 s^−1]	$k_{1,ind}$ ($k_{1,av}$) [×10^-4 s^−1]
1.18 (1.8)	1.34 (1.2)	1.77 (1.8)	2.21 (2.2)	1.72 (1.9)	0.674 (0.59)	1.88 (2.0)

*Compound **2** in presence of 0.9 equivalent of PTSA*

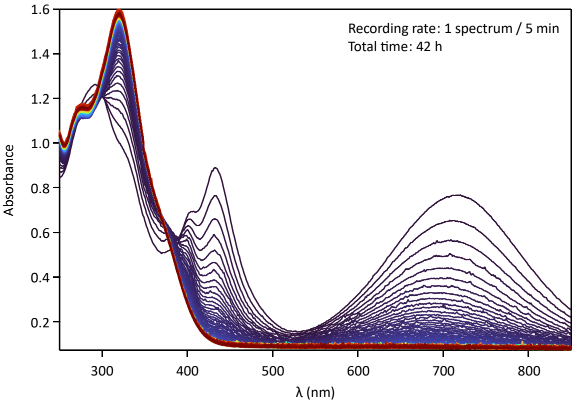

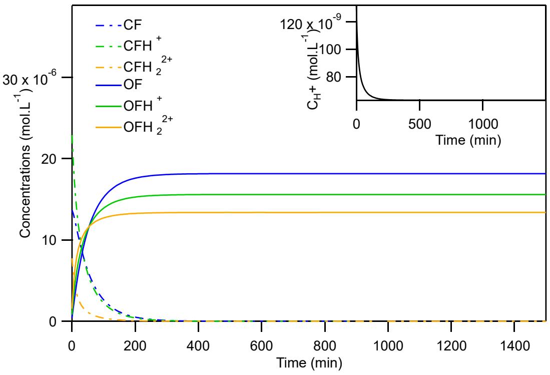

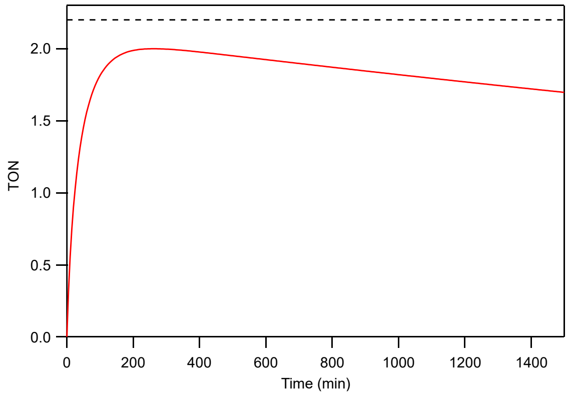

$k_{O1,ind}$ ($k_{O1,av}$) [×10^6 s^−1mol^−1L]	$k_{O-1,ind}$ ($k_{O-1,av}$) [×10^−1 s^−1]	$k_{C1,ind}$ ($k_{C1,av}$) [×10^6 s^−1mol^−1L]	$k_{C-1,ind}$ ($k_{C-1,av}$) [×10^−1 s^−1]	$k_{C2,ind}$ ($k_{C2,av}$) [×10^6 s^−1mol^−1L]	$k_{C-2,ind}$ ($k_{C-2,av}$) [×10^−1 s^−1]	$k_{1,ind}$ ($k_{1,av}$) [×10^-4 s^−1]
1.53 (1.8)	1.13 (1.2)	2.43 (1.8)	1.91 (2.2)	1.93 (1.9)	0.752 (0.59)	2.22 (2.0)

*Compound **2** in presence of 1.5 equivalents of PTSA*

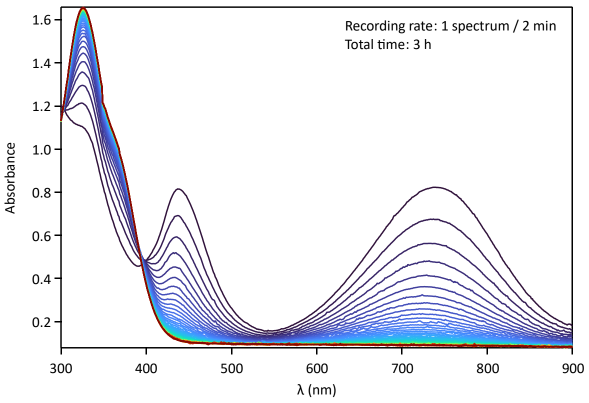

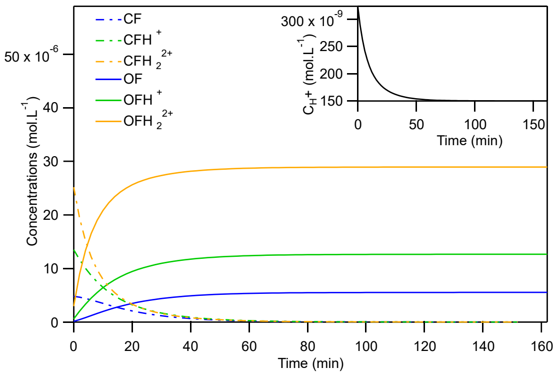

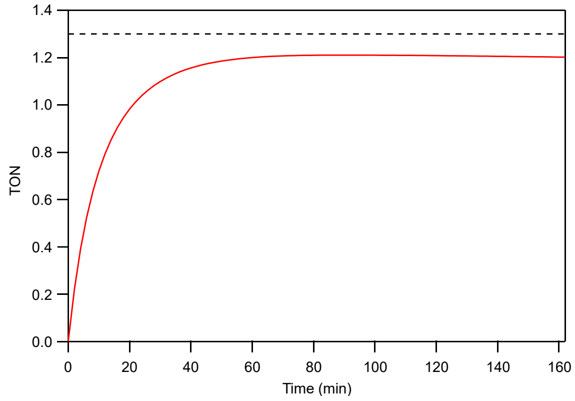

$k_{O1,ind}$ ($k_{O1,av}$) [×10^6 s^{-1}mol^{-1}L]	$k_{O-1,ind}$ ($k_{O-1,av}$) [×10^{-1} s^{-1}]	$k_{C1,ind}$ ($k_{C1,av}$) [×10^6 s^{-1}mol^{-1}L]	$k_{C-1,ind}$ ($k_{C-1,av}$) [×10^{-1} s^{-1}]	$k_{C2,ind}$ ($k_{C2,av}$) [×10^6 s^{-1}mol^{-1}L]	$k_{C-2,ind}$ ($k_{C-2,av}$) [×10^{-1} s^{-1}]	$k_{1,ind}$ ($k_{1,av}$) [×10^{-4} s^{-1}]
2.00 (1.8)	1.31 (1.2)	2.09 (1.8)	2.39 (2.2)	1.83 (1.9)	0.317 (0.59)	1.74 (2.0)

Figure S7: Additional data for acid catalysed back reaction for compounds **1** and **2**.

7. Cyclability experiments

Irradiation at 313 nm in neutral form and spontaneous back reaction in protonated form was performed three times on the sample by successive addition of 1 equivalent (resp. 2) of PTSA (yellow line) and Et_3N (blue line) to a solution of **1** (resp. **2**) in acetonitrile at 20°C.

For practical reasons, in the case of compound **1**, the duration of the thermal back experiment is set at 30 minutes, after which the sample is heated to accelerate the reaction (hatched part) and then cooled to 20°C in order to start again from an initial state rich in OF.

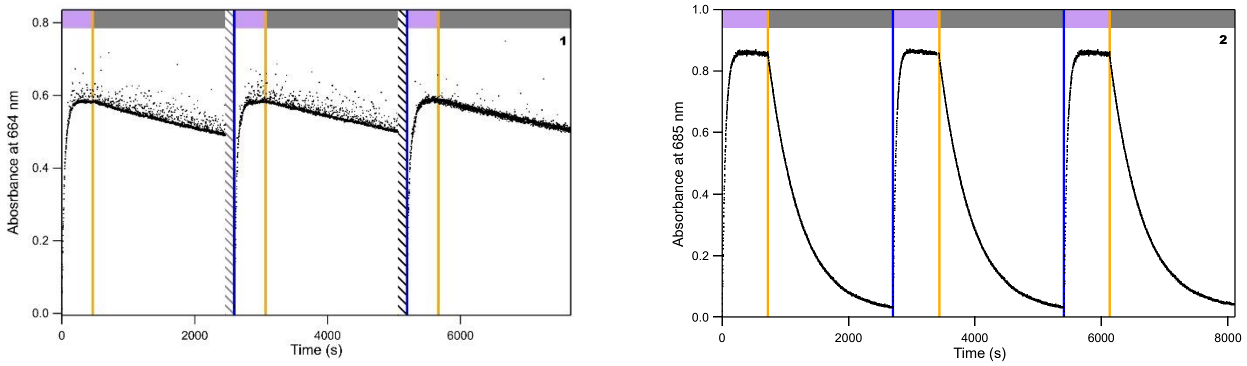

Figure S8: Three cycles of UV irradiation, addition of acid, thermal return and addition of base for **1** (left) and **2** (right).

8. Determination of reaction enthalpies

Micro differential scanning calorimetry (micro-DSC) was conducted using a MICROCALVET ULTRA ® device commercialized by Setaram.

The micro-DSC device is connected to an external temperature control unit filled with water which allows heat transport from the outer layer of the Peltier, optimizing heat control in the micro-DSC. The MICROCALVET apparatus is also connected to a computer which controls the experiment and allows data processing through CALISTO® software. Two different protocols of characterization were designed in the software, both operating in isothermal mode.[8] The protocols are respectively named “thermal return” and “catalytic return” within the frame of this work for reading ease purpose.

Thermal return protocol: the standard cells configuration is used for this protocol. The measurement cell is filled with 500 µL of a solution of **1** or **2** in acetonitrile at its photostationary state (365 nm) while the reference cell is filled with 500 µL of the same solution without irradiation. The temperature of the micro-DSC chamber containing the cells is set at 338 K and the system remains inactive until the heat flow signal stabilization. The two cells are inserted in their dedicated slots and the heat flow signal is registered for 120 minutes, expecting measurement of the heat released during back conversion from the photochrome molecule in the measurement cell.

After the end of experiment, a post-treatment is needed to subtract a heat flow baseline from the measurement, which consists on doing the above-mentioned protocol with acetonitrile containing unconverted photochrome in the measurement cell instead of converted one (reference cell remains identical). This step allows to suppress heat flow variations due to stabilization from the registered measurement.

Catalytic return protocol: the mixing cells configuration is used for this protocol. In this configuration, the measurement and reference cells are divided in two volumes, separated by a polytetrafluoroethylene membrane. Both reference and measurement cell are equipped with a stirring propeller with a needle which allows the perforation of the membrane and then ensure mixing between the upper and lower volume of each cell. Note too that the two stirring propellers are connected to a gear which ensure a simultaneous membrane perforation and homogeneous mixing in both reference and measurement sides.

The lower volume of measurement cell is filled with 150 µL of PTSA solution (C= 6.0 10^{-2} M) while the upper volume is filled with 150 µL of acetonitrile solution containing a defined concentration of

converted photochrome. Both lower part and upper part of reference cell are filled with 75 µL of acid and 75 µL of acetonitrile solution containing unconverted photochrome. Note that the latter mixture has been stirred before introduction in the cell, to achieve the complete protonation of the unconverted photochrome. The temperature of the micro-DSC chamber containing the cells is set at 298 K and the two mixing cells are inserted in their dedicated slots and connected to the mixing gear. The system remains inactive until stabilization of the heat flow signal. The mixing gear is turned on and the heat flow signal is then registered for 120 minutes.

The corresponding data are displayed in Figure S9.

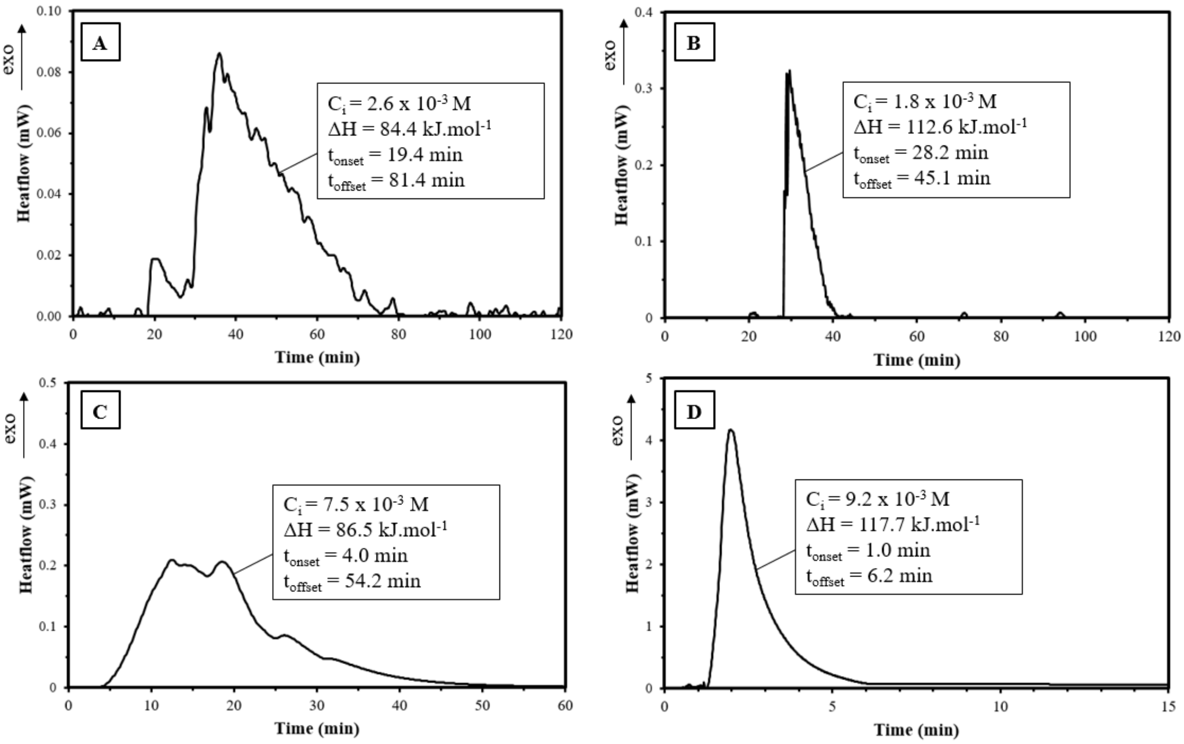

Figure S9: Isothermal mode heat flow analysis of thermal return of **1** (A) and **2** (B) at 338K and catalytic return of **1**·H^+ (C) and **2**·H_2^{2+}(D) at 298K.

9. White light irradiations

In order to simulate solar irradiation, solutions of **1** and **2** were irradiated with a white xenon vapour lamp for 30 minutes, resulting in a conversion of 80% (resp. 82%) of the closed form of **1** (resp. **2**). The associated spectra are shown in Figure S9.

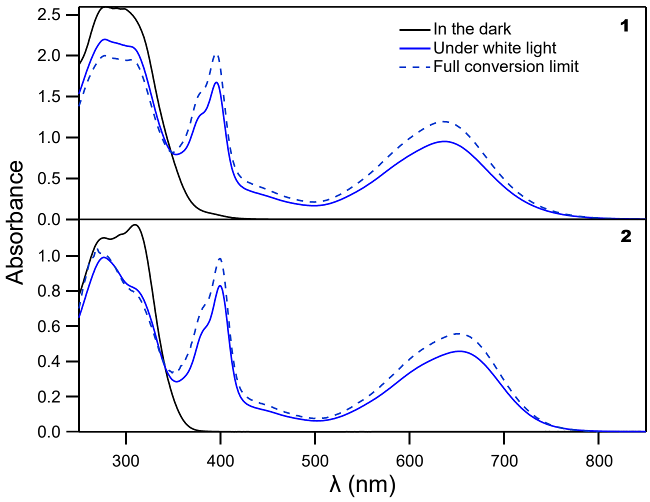

Figure S10: UV-visible spectra of **1** and **2** before and after white light irradiation and the corresponding simulated spectra of the CF.

References

1 V. Valderrey, A. Bonasera, S. Fredrich and S. Hecht, *Angew Chem Int Ed*, 2017, **56**, 1914–1918.
2 S. Qiu, A. T. Frawley, K. G. Leslie and H. L. Anderson, *Chem. Sci.*, 2023, **14**, 9123–9135.
3 N. Baggi, A. Léaustic, S. Groni, E. Anxolabéhère-Mallart, R. Guillot, R. Métivier, F. Maurel and P. Yu, *Chem. Eur. J.*, 2021, **27**, 12866–12876.
4 M. Schnürch, J. Hämmerle, M. Mihovilovic and P. Stanetty, *Synthesis*, 2010, **2010**, 837–843.
5 J. Hämmerle, M. Spina, M. Schnürch, M. Mihovilovic and P. Stanetty, *Synthesis*, 2008, **2008**, 3099–3107.
6 Y. Hashimoto, T. Nakashima, D. Shimizu and T. Kawai, *Chem. Commun.*, 2016, **52**, 5171–5174.
7 T. Yanai, D. P. Tew and N. C. Handy, *Chemical Physics Letters*, 2004, **393**, 51–57.
8 G. W. H. Höhne, W. F. Hemminger and H.-J. Flammersheim, *Differential Scanning Calorimetry*, Springer Berlin Heidelberg, Berlin, Heidelberg, 2003.
